# Supplementary material for: Unsustainable anthropogenic mortality threatens the long-term viability of lion populations in Mozambique
Source: PLoS One. 2025 Jun 10;20(6):e0325745. doi: 10.1371/journal.pone.0325745 (PMC12151417; doi:10.1371/journal.pone.0325745)
Supplement: S1 File — S1 Appendix. Descriptions of lion populations in Mozambique. S2 Appendix. Population modelling details. S1 Table. Tukey post-hoc test results derived from the multinomial linear regression models. S2 Table. Details of estimated detection rates, anthropogenic mortality rates (AMR) and recovery targets for lion populations within Mozambique. Included are current AMRs, trends in AMRs, and targets for AMR reduction needed to promote lion recovery. Effects of interventions on AMRs are also provided to highlight the importance of monitoring and veterinary capacity. (DOCX) [file pone.0325745.s001.docx]

Unsustainable anthropogenic mortality threatens the long-term viability of lion populations in Mozambique

**João Almeida, Willem Briers-Louw, Agostinho Jorge, Colleen Begg, Marnus Roodbol, Hans Bauer,** **Andrew Loveridge, Matthew Wijers, Rob Slotow, Peter Lindsey, Kristoffer Everatt, Holly Rosier, Sean Nazerali, Lizanne Roxburgh, Hugo Pereira, Mercia da Conceicao, Armindo Araman, Osvaldo J. Abrao, Alison J. Leslie, Franziska Steinbruch, Vincent N. Naude, Samantha K. Nicholson**

**Supplementary Material**

**Appendix S1. Descriptions of lion populations in Mozambique.**

1. **Northern Mozambique**
   1. **Niassa Special Reserve (42,353 km^2^)**

Niassa Special Reserve (SR), located in northern Mozambique, is a vast protected area (PA) that represents one of only eight lion populations in Africa exceeding 1,000 individuals (Riggio et al., 2012). This reserve is administratively divided into 17 management blocks allocated for ecotourism and sport hunting. The total area designated for hunting covers 27,986 km^2^, with hunting revenue contributing approximately one third of the reserves’ annual operational budget, including funds for anti-poaching and overall management (Jorge et al., 2013). Since 2012, Niassa has been co-managed by Mozambique’s National Administration for Conservation Areas (ANAC) and the Wildlife Conservation Society, following its previous management by the Sociedade de Gestão e Desenvolvimento da Reserva do Niassa (SRN). Sport hunting of lions has been regulated since 2004 though a points system, implemented by the Niassa Carnivore Project, a field-based NGO. This system has effectively alleviated pressure on the lion population, contributing to its protection (Begg et al., 2018). While the core areas of Niassa SR are well protected, much of the peripheries are inadequately protected, and over a decade of monitoring has revealed substantial illegal killing of lions, which has adversely affected the population (Niassa Carnivore Programme, 2023). For example, between 2017 and 2023 alone, at least 115 lions were reported to have been illegally killed (Niassa Carnivore Programme, 2023). The primary threat to lions in this landsape is bushmeat by-catch, although there is increasing evidence of targeted poaching for lion body parts, which raises major concerns regarding this significant lion population.

1. **Central Mozambique**
   1. **Gorongosa National Park (3,708 km^2^)**

Armed conflicts during the Mozambican Civil War (1977–1992) were particularly concentrated around Gorongosa National Park (Hatton et al. 2001). These conflicts, coupled with unregulated and pervasive bushmeat poaching resulted in dramatic wildlife population declines, with some species such as leopards (*Panthera pardus*) and spotted hyaenas (*Crocuta crocuta*) becoming extirpated within the park. In response to these challenges, a long-term public-private initiative was initiated in 2007 with the aim of restoring wildlife populations, the ecotourism market, and livelihoods of communities living in and around the NP (Pringle, 2017). Over time, significant recovery of prey populations was observed, facilitate by improved management and enhanced protection efforts (Stalmans et al., 2019). However, little was known about the remaining lion population at that stage, the only remaining large carnivore in the park. In 2012, intensive monitoring of this population commenced through collaring and monitoring of lions, as well implementing veterinary intervention practices where needed (Bouley et al., 2018). By 2016, the lion population had increased to over 100 individuals (Bouley et al., 2018), and it is currently estimated to exceed 180 individuals. There is emerging evidence of lion movement into the nearby Zambezi Delta (Antonio Paulo, Gorongosa NP, pers. comm.), although the extent and dynamics of this connectivity remains insufficiently understood and requires further investigation. Gorongosa boasts the highest operational capacity for lion conservation in Mozambique, with a large annual budget ($9 million in 2017; Pringle, 2017), and employment of approximately 300 rangers, which positions the park as a critical site for lion population recovery in the region. Despite this, bushmeat by-catch remains an important threat to the lion population, with isolated cases reported annually.

- 1. **Coutadas 9/13 (9,724 km^2^)**

The lion population in Coutadas 9 and 13 was severely depleted due to decades of armed conflict and persistent bushmeat poaching (Lindsey & Bento, 2012). While occasional sightings of transient individuals were reported, by the late 2000s, the population was considered functionally extirpated. In 2010, management of Coutada 9 initiated the reintroduction of ten lions with the aim of facilitating population recovery and generating additional revenue to sustain the WMAs. These lions, sourced from a private game reserve in South Africa, where they occurred in surplus, were considered ideal candidates for reintroduction. Following the reintroduction, the population displayed initial population growth, supported by abundant prey and a reduction in bushmeat poaching to more manageable levels (Lindsey & Bento, 2012). Regular monitoring of the population, including the use of satellite collars, and veterinary interventions were conducted in response to lions caught in steel gin (or ‘bear-claw’) traps, the main form of poaching in these WMAs (﻿Lindsey et al., 2013), helped to enhance survival rates. Today, the population remains under pressure largely due to rising incidents of lion mortalities, primarily linked to bushmeat by-catch, with some evidence of targeted poaching for body parts. Coutada 9 is allocated a quota of two lions annually for trophy hunting, although actual offtake is occasionally lower than quota (e.g., in 2024 where only one lion was taken). This relatively small, regulated offtake helps to generate an essential proportion (~8%) of the total revenue for this WMA, which is critical for funding anti-poaching efforts and broader conservation efforts (Lindsey & Bento, 2012). In contrast, Coutada 13, adjacent to Coutada 9, has not been formally managed for several years due to complications around government-lease agreements, a situation mirrored by other vacant concessions across the country. Despite this lack of formal management, Coutada 13 remains an important area for lion conservation, both for its large size and for its role in supporting seasonal movements of lion and their prey from Coutada 9. Given the uncontrolled expansion of human settlements within Coutada 13, it is imperative that this WMA undergoes immediate and effective management to preserve this crucial habitat for lions and their prey. Without such intervention, the area risks becoming a sink for the lion population, undermining broader recovery efforts.

- 1. **Zambezi Delta (9,750 km^2^)**

Historically lions were abundant in the Zambezi Delta (Maugham, 1914; Smithers & Tello, 1976), but the decades of intense armed conflict in the region, compounded by sustained bushmeat poaching, led to the functional extirpation of the species by the mid-2010s. However, following the end of the conflict and with enhance protection efforts, the ecosystem’s high productivity facilitated the rapid recovery of prey populations, creating favourable conditions for the potential recovery of large carnivores (Briers-Louw et al., 2024). Since natural recolonization by lions was deemed unlikely, a decision was made to reintroduce the species in 2018. A total of 24 lions, sourced from various private game reserves in South Africa, where they occurred in excess, were selected as ideal candidates for reintroduction. The reintroduced lions displayed successful range residency, reproduction and initial population growth. To further support population growth and genetic diversity, the population has occasionally been augmented with male lions from southern Mozambique. Despite these positive developments, the population continues to face significant threats from bushmeat by-catch, as well as some evidence of targeted poaching for lion body parts. These pressures may continue to suppress the population well below ecological carrying capacity (Briers-Louw et al., 2024). Ecologically, the Zambezi Delta could several hundred lions, with the prospect of becoming a self-sustaining lion stronghold, especially if connectivity with nearby Gorongosa NP is successfully established.

- 1. **Tchuma Tchato (38,000 km^2^)**

The Tchuma Tchato Community Programme (TTCP) in Tete Province historically supported a significant lion population (Smithers & Tello, 1976). However, this landscape remains largely data-deficient regarding current lion population estimates and the threats that impact them. Early population estimates for the region ranged from 125 to 507 lions (Bauer & van der Merwe, 2004; Chardonnet et al., 2002; Chardonnet et al., 2009), with the most recent estimate suggesting around 180 lions within approximately two-thirds of the region (Jacobson et al., 2013). While these numbers appear promising, the study primarily relied on indirect data collection methods, such as interviews and extrapolations based on presumed habitat availability, which have been increasingly criticized for their tendency to overestimate population sizes (**﻿**Norton, 1990). Consequently, these estimates were excluded from our study due to concerns about their reliability. Nevertheless, these estimates provide an indication of another key lion population in Mozambique, which requires an urgent need for more accurate and systematic monitoring. Similar to the situation in Limpopo NP, which is largely supported by the Kruger NP lion population, it is hypothesized that the TTCP lion population may be supported by transient lions from Zambia and Zimbabwe (Jacobson et al., 2013). However, due to the absence of targeted lion monitoring, the expansion of human settlements, and the likely high levels of anthropogenically mortality (patterns observed in better-studied populations within the country), it is probable that the TTCP lion population has experienced further population size reductions.

1. **Southern Mozambique**
   1. **Limpopo National Park (10,544 km^2^)**

Limpopo National Park, the largest NP in Mozambique, was officially established in 2001 and forms an important part of the Greater Limpopo Transfrontier Park (GLTFP), which also includes Kruger NP in South Africa. The park is co-managed by Mozambique’s National Administration of Conservation Areas (ANAC) and Peace Parks Foundation. During the prolonged periods of the armed conflict in Mozambique, wildlife populations within the park were severely depleted. However, as part of the GLTFP initiative, a section of the border was opened to facilitate the nature recolonization of wildlife species from Kruger NP into Limpopo NP (Hanks, 2000). Despite over two decades of post-war restoration efforts and evidence of population increases for certain species, the overall recovery of wildlife populations within the park remains in a nascent and vulnerable state (Roque et al., 2018). This process continues to be impeded by ongoing anthropogenic pressures, including livestock grazing and bushmeat poaching (Everatt et al., 2019). Additionally, several large communities occupy internation areas of the park, reducing its effective size and complicating conservation efforts. Over just a five-year period, the lion population in Limpopo NP experienced a dramatic 66% decline (Everatt et al., 2019). The main causes of lion mortality have been identified as bushmeat by-catch, targeted lion poaching, and retaliation. A particularly alarming trend is the increasing removal of lion body parts, such as teeth and paws, to fuel the illegal wildlife trade. Of particular concern is the widespread use of lethal poisons for targeted lion killings, which presents a growing threat to both the lion population and broader biodiversity within the park (Everatt et al., 2019). These ongoing pressures underscore the urgent need for enhanced management strategies and doubling down on anti-poaching measures to safeguard the long-term viability of lions and other wildlife in the NP.

- 1. **Greater Lebombo Conservancy (2,483 km^2^)**

The Greater Lebombo Conservancy (GLC) is a private, securitized conservancy which was established in 2012 to serve as a buffer zone aimed at restricting cross-border movement of commercial rhino poachers (Massé & Lunstrum, 2016). The GLC comprises four conservation areas: Karingani Game Reserve, Sabie Game Park, Massintono Concession, Mbhatse Concession. Collectively, these areas support both non-consumptive and hunting-related safari operations (Massé & Lunstrum, 2016), and are independently enclosed by predator-proof fencing, with Karingani GR and Sabie GP having open boundaries with Kruger NP. These areas are well-managed and protected, with ongoing wildlife management efforts like ungulate restocking as required and predator offtake (through translocations) to reduce pressure on prey populations. Currently, an estimated 70 lions occur across the GLC, although effective monitoring remains challenging due to movement of lions into Kruger NP.

- 1. **Zinave NP (4,080 km^2^; Sanctuary 186 km^2^)**

Zinave National Park covers approximately 4,080 km^2^ and forms a key part of the Great Limpopo Transfrontier Conservation Area (GLTFCA). Originally gazetted as a hunting concession in the 1960s, the NP was officially declared a PA in 1973. Like many other conservation areas in Mozambique, Zinave suffered from decades of conflict during the Mozambican Civil War and subsequent poaching resulted in the decimation of wildlife populations. In 2015, a 20-year co-management agreement was signed between ANAC and the Peace Parks Foundation. This partnership marked the beginning of substantial restoration efforts within the NP, which initially focused on introducing rangers to reduce the impact of bushmeat poaching (Peace Parks Foundation, 2023). Subsequently, a 186 km^2^ fenced ‘sanctuary’, serving as the core protection zone was constructed, within which over 2,500 game animals were reintroduced. Between 2020 and 2021, four spotted hyenas and two leopards were also reintroduced into the park as part of the ecological restoration process. In 2021, after an absence of nearly two decades, the first lion was sighted in Zinave. It is believed that the high prey biomass within the sanctuary helped anchored this individual. Since then, an additional five individuals have settled in the park, suggesting a natural recolonization process likely facilitated by dispersing individuals from within the GLTFCA. This small population is currently monitored by onsite researchers. However, challenges persist. In 2024, one of the male lions was caught in a wire bushmeat snare, and rapid veterinary intervention allowed for the removal of the snare and treatment of the wound by veterinarians, highlighting both the susceptibility of lions to snaring and the importance of rapid intervention capacity (Peace Parks Foundation, 2023). Additionally, given the small NP size and presence of fencing, this population will required ongoing management if it is to become a potential source location for lions and thus contribute towards lion conservation in Mozambique.

1. **Outside Protected Areas**

Mozambique is a large country (800,000 km^2^) with ~29% (or 233,249 km^2^) of the terrestrial landmass gazetted for conservation (i.e., PAs and WMAs). While the vast majority of the country’s lion population occur within these designated conservation areas, lions are occasionally observed moving into non-protected areas across the country (Chardonnet et al., 2009). Such incidents most often involve the dispersal of relatively young lions searching for vacant territories or damage-causing animals that escape or venture beyond the boundaries of protected zones. Such movements are not uncommon, particularly given that most conservation areas across the country are unfenced, allowing lions to roam outside protected areas. Where these individuals are involved in human-wildlife conflict, the national wildlife authority (ANAC) is alerted. ANAC’s response dependent on the circumstance of each incident. For instance, if lions are considered DCAs due to repeat-offenses, these individuals are either euthanised or translocated a considerable distance from their source location. Conversely, lions involved in isolated incidents are typically relocated back into their original conservation areas if possible. The fate of lions moving beyond conservation areas largely depends on the human density of the areas they traverse and the prevailing attitudes towards wildlife. In some cases, lions may find suitable habitats, while in others, they may continue to experience conflict with local communities. This highlights the ongoing challenges of managing lion populations in unfenced landscapes and the complex interactions between conservation efforts and human-wildlife conflict.

**Appendix S2. Population modelling details.**

Universal parameters:

Runs = 1000

Survival = Loveridge et al. 2023

Litter size distribution = (0.12, 0.30, 0.35, 0.19, 0.04) [for sizes 1-5]

Initial population demographics = (0.25, 0.30, 0.40, 0.77) [for sub-adult 3-4, sub-adult 2-3, yearlings and cubs]

Initial sex ratios = (0.5, 0.5, 0.5, 0.5) [for sub-adult 3-4, sub-adult 2-3, yearlings and cubs]

Conflict age threshold = 0 years

Hunting age threshold = 6 years

Without accurate data on home range size, maximum number of prides and coalitions was calculated as follows:

Formula A:

$$N = \frac{C - (0.05 \times C)}{12}$$

Where N is maximum number of prides or coalitions that can fit in the area and C is carrying capacity. This is based on the assumption that 5% of the population at carrying capacity are vagrants and the rest are in prides. We then divide by average pride size at carrying capacity (12) [[Smuts 1976]](https://www.researchgate.net/publication/274660318_Population_characteristics_and_recent_history_of_lions_in_two_parts_of_Kruger_National_Park) [[Nams et al. 2023]](https://onlinelibrary.wiley.com/doi/full/10.1111/mam.12309)

Site-specific parameters:

**Limpopo + K (Kruger National Park)**

| Parameter | Value | Source |
| --- | --- | --- |
| Carrying Capacity | 850 | Estimated by authors - approx. double the CC of LNP^1^ |
| Max number of prides | 67 | Formula A |
| Max number of coalitions | 67 | Formula A |
| Number of prides vulnerable to AM | 34 (50%) | Only LNP assumed to be vulnerable to AM.^1^ |

**Limpopo – K (Kruger National Park)**

| Parameter | Value | Source |
| --- | --- | --- |
| Carrying Capacity | 438 | PA rep. |
| Max number of prides | 34 | Formula A |
| Max number of coalitions | 34 | Formula A |
| Number of prides vulnerable to AM | 34 (100%) |  |

**Gorongosa**

| Parameter | Value | Source |
| --- | --- | --- |
| Carrying Capacity | 468 | PA rep. |
| Max number of prides | 37 | Formula A |
| Max number of coalitions | 37 | Formula A |
| Number of prides vulnerable to AM | 37 (100%) |  |

**Coutadas 9 and 13**

| Parameter | Value | Source |
| --- | --- | --- |
| Carrying Capacity | 220 | PA rep. |
| Max number of prides | 17 | Formula A |
| Max number of coalitions | 17 | Formula A |
| Number of prides vulnerable to AM | 17 (100%) |  |

**Zambezi Delta**

| Parameter | Value | Source |
| --- | --- | --- |
| Carrying Capacity | 663 | PA rep. |
| Max number of prides | 55 | Formula A |
| Max number of coalitions | 55 | Formula A |
| Number of prides vulnerable to AM | 55 (100%) |  |

**Niassa**

| Parameter | Value | Source |
| --- | --- | --- |
| Carrying Capacity | 1912 | PA rep. |
| Max number of prides | 151 | Formula A |
| Max number of coalitions | 151 | Formula A |
| Number of prides vulnerable to AM | 125 (83%) | 17% Niassa is well protected [PA rep].^2^ |

^1^Limpopo Modelling

As Limpopo National Park is connected to Kruger National Park along most of its western boundary, we created a model that combined these two areas to reflect the source - sink dynamic. Northern Kruger was defined as the protected core area and LNP was defined as the vulnerable edge zone. Northern Kruger is approximately the same size as LNP, we therefore set the carrying capacity of the combined area as 850 (approx double the CC of LNP alone) and the protected:vulnerable pride ratio at 50:50.

^2^Niassa Modelling

We accounted for a source - sink dynamic in Niassa as 17% of the reserve is well protected. We therefore set the protected:vulnerable pride ratio to 17:83.

**Table S1. Tukey post-hoc test results derived from the multinomial linear regression models.**

(A) Temporal differences by motive and method. Presented are *P*-values, with significance (*P* < 0.05) highlighted in bold.

| Motive = Accidental | | | | | | | | | | | | | | |
| --- | --- | --- | --- | --- | --- | --- | --- | --- | --- | --- | --- | --- | --- | --- |
| Year | 2010 | 2011 | 2012 | 2013 | 2014 | 2015 | 2016 | 2017 | 2018 | 2019 | 2020 | 2021 | 2022 | 2023 |
| 2010 | 0.0000 | 1.0000 | 1.0000 | 1.0000 | 1.0000 | 1.0000 | 1.0000 | 0.9990 | 1.0000 | 1.0000 | 1.0000 | 1.0000 | 1.0000 | 1.0000 |
| 2011 | 1.0000 | 0.0000 | 1.0000 | 1.0000 | 1.0000 | 1.0000 | 1.0000 | 0.9990 | 1.0000 | 1.0000 | 1.0000 | 1.0000 | 1.0000 | 1.0000 |
| 2012 | 1.0000 | 1.0000 | 0.0000 | 1.0000 | 1.0000 | 1.0000 | 1.0000 | 0.9990 | 1.0000 | 1.0000 | 1.0000 | 1.0000 | 1.0000 | 1.0000 |
| 2013 | 1.0000 | 1.0000 | 1.0000 | 0.0000 | 1.0000 | 1.0000 | 1.0000 | 0.9990 | 1.0000 | 1.0000 | 1.0000 | 1.0000 | 1.0000 | 1.0000 |
| 2014 | 1.0000 | 1.0000 | 1.0000 | 1.0000 | 0.0000 | 1.0000 | 1.0000 | 0.9990 | 1.0000 | 1.0000 | 1.0000 | 1.0000 | 1.0000 | 1.0000 |
| 2015 | 1.0000 | 1.0000 | 1.0000 | 1.0000 | 1.0000 | 0.0000 | 1.0000 | 0.9990 | 1.0000 | 1.0000 | 1.0000 | 1.0000 | 1.0000 | 1.0000 |
| 2016 | 1.0000 | 1.0000 | 1.0000 | 1.0000 | 1.0000 | 1.0000 | 0.0000 | 0.9990 | 1.0000 | 1.0000 | 1.0000 | 1.0000 | 1.0000 | 1.0000 |
| 2017 | 0.9990 | 0.9990 | 0.9990 | 0.9990 | 0.9990 | 0.9990 | 0.9990 | 0.0000 | 1.0000 | 1.0000 | 1.0000 | 1.0000 | 1.0000 | 1.0000 |
| 2018 | 1.0000 | 1.0000 | 1.0000 | 1.0000 | 1.0000 | 1.0000 | 1.0000 | 1.0000 | 0.0000 | 1.0000 | 1.0000 | 1.0000 | 1.0000 | 1.0000 |
| 2019 | 1.0000 | 1.0000 | 1.0000 | 1.0000 | 1.0000 | 1.0000 | 1.0000 | 1.0000 | 1.0000 | 0.0000 | 1.0000 | 1.0000 | 1.0000 | 1.0000 |
| 2020 | 1.0000 | 1.0000 | 1.0000 | 1.0000 | 1.0000 | 1.0000 | 1.0000 | 1.0000 | 1.0000 | 1.0000 | 0.0000 | 1.0000 | 1.0000 | 1.0000 |
| 2021 | 1.0000 | 1.0000 | 1.0000 | 1.0000 | 1.0000 | 1.0000 | 1.0000 | 1.0000 | 1.0000 | 1.0000 | 1.0000 | 0.0000 | 1.0000 | 1.0000 |
| 2022 | 1.0000 | 1.0000 | 1.0000 | 1.0000 | 1.0000 | 1.0000 | 1.0000 | 1.0000 | 1.0000 | 1.0000 | 1.0000 | 1.0000 | 0.0000 | 1.0000 |
| 2023 | 1.0000 | 1.0000 | 1.0000 | 1.0000 | 1.0000 | 1.0000 | 1.0000 | 1.0000 | 1.0000 | 1.0000 | 1.0000 | 1.0000 | 1.0000 | 0.0000 |
| Motive = Bushmeat bycatch | | | | | | | | | | | | | | |
| Year | 2010 | 2011 | 2012 | 2013 | 2014 | 2015 | 2016 | 2017 | 2018 | 2019 | 2020 | 2021 | 2022 | 2023 |
| 2010 | 0.0000 | 1.0000 | 1.0000 | 1.0000 | 1.0000 | 1.0000 | 0.5242 | 1.0000 | 0.3118 | 0.9999 | 0.7566 | 1.0000 | 1.0000 | 0.4166 |
| 2011 | 1.0000 | 0.0000 | 1.0000 | 1.0000 | 1.0000 | 1.0000 | 0.8043 | 1.0000 | 0.5678 | 1.0000 | 0.9375 | 0.9999 | 1.0000 | 0.7117 |
| 2012 | 1.0000 | 1.0000 | 0.0000 | 1.0000 | 1.0000 | 1.0000 | 0.5242 | 1.0000 | 0.3118 | 0.9999 | 0.7566 | 1.0000 | 1.0000 | 0.4165 |
| 2013 | 1.0000 | 1.0000 | 1.0000 | 0.0000 | 1.0000 | 1.0000 | 0.1472 | 1.0000 | 0.0791 | 0.9930 | 0.3849 | 1.0000 | 1.0000 | 0.0892 |
| 2014 | 1.0000 | 1.0000 | 1.0000 | 1.0000 | 0.0000 | 1.0000 | 0.5147 | 1.0000 | 0.3003 | 1.0000 | 0.7834 | 0.9998 | 1.0000 | 0.3916 |
| 2015 | 1.0000 | 1.0000 | 1.0000 | 1.0000 | 1.0000 | 0.0000 | 0.2209 | 1.0000 | 0.1198 | 0.9993 | 0.5122 | 1.0000 | 1.0000 | 0.1388 |
| 2016 | 0.5242 | 0.8043 | 0.5242 | 0.1472 | 0.5147 | 0.2209 | 0.0000 | 0.1746 | 1.0000 | 0.8777 | 1.0000 | **0.0411** | 0.3890 | 1.0000 |
| 2017 | 1.0000 | 1.0000 | 1.0000 | 1.0000 | 1.0000 | 1.0000 | 0.1746 | 0.0000 | 0.0951 | 0.9982 | 0.4479 | 1.0000 | 1.0000 | 0.1053 |
| 2018 | 0.3118 | 0.5678 | 0.3118 | 0.0791 | 0.3003 | 0.1198 | 1.0000 | 0.0951 | 0.0000 | 0.6470 | 1.0000 | **0.0249** | 0.2174 | 1.0000 |
| 2019 | 0.9999 | 1.0000 | 0.9999 | 0.9930 | 1.0000 | 0.9993 | 0.8777 | 0.9982 | 0.6470 | 0.0000 | 0.9772 | 0.9265 | 1.0000 | 0.7882 |
| 2020 | 0.7566 | 0.9375 | 0.7566 | 0.3849 | 0.7834 | 0.5122 | 1.0000 | 0.4479 | 1.0000 | 0.9772 | 0.0000 | 0.1741 | 0.7077 | 1.0000 |
| 2021 | 1.0000 | 0.9999 | 1.0000 | 1.0000 | 0.9998 | 1.0000 | **0.0411** | 1.0000 | **0.0249** | 0.9265 | 0.1741 | 0.0000 | 0.9957 | **0.0208** |
| 2022 | 1.0000 | 1.0000 | 1.0000 | 1.0000 | 1.0000 | 1.0000 | 0.3890 | 1.0000 | 0.2174 | 1.0000 | 0.7077 | 0.9957 | 0.0000 | 0.2685 |
| 2023 | 0.4166 | 0.7117 | 0.4165 | 0.0892 | 0.3916 | 0.1388 | 1.0000 | 0.1053 | 1.0000 | 0.7882 | 1.0000 | **0.0208** | 0.2685 | 0.0000 |
| Motive = DCA | | | | | | | | | | | | | | |
| Year | 2010 | 2011 | 2012 | 2013 | 2014 | 2015 | 2016 | 2017 | 2018 | 2019 | 2020 | 2021 | 2022 | 2023 |
| 2010 | 0.0000 | 1.0000 | 1.0000 | 1.0000 | 1.0000 | 1.0000 | 1.0000 | 1.0000 | 1.0000 | 1.0000 | 1.0000 | 1.0000 | 0.8678 | 1.0000 |
| 2011 | 1.0000 | 0.0000 | 1.0000 | 1.0000 | 1.0000 | 1.0000 | 1.0000 | 1.0000 | 1.0000 | 1.0000 | 1.0000 | 1.0000 | 0.8678 | 1.0000 |
| 2012 | 1.0000 | 1.0000 | 0.0000 | 1.0000 | 1.0000 | 1.0000 | 1.0000 | 1.0000 | 1.0000 | 1.0000 | 1.0000 | 1.0000 | 0.8678 | 1.0000 |
| 2013 | 1.0000 | 1.0000 | 1.0000 | 0.0000 | 1.0000 | 1.0000 | 1.0000 | 1.0000 | 1.0000 | 1.0000 | 1.0000 | 1.0000 | 0.8678 | 1.0000 |
| 2014 | 1.0000 | 1.0000 | 1.0000 | 1.0000 | 0.0000 | 1.0000 | 1.0000 | 1.0000 | 1.0000 | 1.0000 | 1.0000 | 1.0000 | 0.8678 | 1.0000 |
| 2015 | 1.0000 | 1.0000 | 1.0000 | 1.0000 | 1.0000 | 0.0000 | 1.0000 | 1.0000 | 1.0000 | 1.0000 | 1.0000 | 1.0000 | 0.8678 | 1.0000 |
| 2016 | 1.0000 | 1.0000 | 1.0000 | 1.0000 | 1.0000 | 1.0000 | 0.0000 | 1.0000 | 1.0000 | 1.0000 | 1.0000 | 1.0000 | 0.8678 | 1.0000 |
| 2017 | 1.0000 | 1.0000 | 1.0000 | 1.0000 | 1.0000 | 1.0000 | 1.0000 | 0.0000 | 1.0000 | 1.0000 | 1.0000 | 1.0000 | 0.8678 | 1.0000 |
| 2018 | 1.0000 | 1.0000 | 1.0000 | 1.0000 | 1.0000 | 1.0000 | 1.0000 | 1.0000 | 0.0000 | 1.0000 | 1.0000 | 1.0000 | 0.8678 | 1.0000 |
| 2019 | 1.0000 | 1.0000 | 1.0000 | 1.0000 | 1.0000 | 1.0000 | 1.0000 | 1.0000 | 1.0000 | 0.0000 | 1.0000 | 1.0000 | 0.8678 | 1.0000 |
| 2020 | 1.0000 | 1.0000 | 1.0000 | 1.0000 | 1.0000 | 1.0000 | 1.0000 | 1.0000 | 1.0000 | 1.0000 | 0.0000 | 1.0000 | 0.8678 | 1.0000 |
| 2021 | 1.0000 | 1.0000 | 1.0000 | 1.0000 | 1.0000 | 1.0000 | 1.0000 | 1.0000 | 1.0000 | 1.0000 | 1.0000 | 0.0000 | 0.8678 | 1.0000 |
| 2022 | 0.8678 | 0.8678 | 0.8678 | 0.8678 | 0.8678 | 0.8678 | 0.8678 | 0.8678 | 0.8678 | 0.8678 | 0.8678 | 0.8678 | 0.0000 | 0.8678 |
| 2023 | 1.0000 | 1.0000 | 1.0000 | 1.0000 | 1.0000 | 1.0000 | 1.0000 | 1.0000 | 1.0000 | 1.0000 | 1.0000 | 1.0000 | 0.8678 | 0.0000 |
| Motive = Retaliation | | | | | | | | | | | | | | |
| Year | 2010 | 2011 | 2012 | 2013 | 2014 | 2015 | 2016 | 2017 | 2018 | 2019 | 2020 | 2021 | 2022 | 2023 |
| 2010 | 0.0000 | 1.0000 | 0.9984 | 0.9664 | 0.8318 | 0.4652 | 0.9990 | 0.4743 | 0.9989 | 0.9990 | 0.2108 | 0.1459 | 0.8678 | 1.0000 |
| 2011 | 1.0000 | 0.0000 | 0.9984 | 0.9664 | 0.8318 | 0.4652 | 0.9990 | 0.4743 | 0.9989 | 0.9990 | 0.2108 | 0.1459 | 0.8678 | 1.0000 |
| 2012 | 0.9984 | 0.9984 | 0.0000 | 1.0000 | 1.0000 | 1.0000 | 1.0000 | 1.0000 | 1.0000 | 1.0000 | 0.9967 | 0.9992 | 1.0000 | 0.9984 |
| 2013 | 0.9664 | 0.9664 | 1.0000 | 0.0000 | 1.0000 | 1.0000 | 0.9999 | 1.0000 | 1.0000 | 0.9999 | 0.9537 | 0.9752 | 1.0000 | 0.9664 |
| 2014 | 0.8318 | 0.8318 | 1.0000 | 1.0000 | 0.0000 | 1.0000 | 0.9901 | 1.0000 | 0.9989 | 0.9900 | 0.9992 | 0.9999 | 0.9999 | 0.8318 |
| 2015 | 0.4652 | 0.4652 | 1.0000 | 1.0000 | 1.0000 | 0.0000 | 0.9369 | 1.0000 | 0.9917 | 0.9369 | 0.9990 | 0.9999 | 0.9992 | 0.4652 |
| 2016 | 0.9990 | 0.9990 | 1.0000 | 0.9999 | 0.9901 | 0.9369 | 0.0000 | 0.9526 | 1.0000 | 1.0000 | 0.5467 | 0.5323 | 1.0000 | 0.9990 |
| 2017 | 0.4743 | 0.4743 | 1.0000 | 1.0000 | 1.0000 | 1.0000 | 0.9526 | 0.0000 | 0.9953 | 0.9526 | 0.9974 | 0.9995 | 0.9997 | 0.4743 |
| 2018 | 0.9989 | 0.9989 | 1.0000 | 1.0000 | 0.9989 | 0.9917 | 1.0000 | 0.9953 | 0.0000 | 1.0000 | 0.7382 | 0.7653 | 1.0000 | 0.9989 |
| 2019 | 0.9990 | 0.9990 | 1.0000 | 0.9999 | 0.9900 | 0.9369 | 1.0000 | 0.9526 | 1.0000 | 0.0000 | 0.5467 | 0.5323 | 1.0000 | 0.9990 |
| 2020 | 0.2108 | 0.2108 | 0.9967 | 0.9537 | 0.9992 | 0.9990 | 0.5467 | 0.9974 | 0.7382 | 0.5467 | 0.0000 | 1.0000 | 0.8446 | 0.2108 |
| 2021 | 0.1459 | 0.1459 | 0.9992 | 0.9752 | 0.9999 | 0.9999 | 0.5323 | 0.9995 | 0.7653 | 0.5323 | 1.0000 | 0.0000 | 0.8740 | 0.1459 |
| 2022 | 0.8678 | 0.8678 | 1.0000 | 1.0000 | 0.9999 | 0.9992 | 1.0000 | 0.9997 | 1.0000 | 1.0000 | 0.8446 | 0.8740 | 0.0000 | 0.8678 |
| 2023 | 1.0000 | 1.0000 | 0.9984 | 0.9664 | 0.8318 | 0.4652 | 0.9990 | 0.4743 | 0.9989 | 0.9990 | 0.2108 | 0.1459 | 0.8678 | 0.0000 |
| Motive = Targeted poaching | | | | | | | | | | | | | | |
| Year | 2010 | 2011 | 2012 | 2013 | 2014 | 2015 | 2016 | 2017 | 2018 | 2019 | 2020 | 2021 | 2022 | 2023 |
| 2010 | 0.0000 | 0.9987 | 0.9984 | 0.9664 | 0.9989 | 0.1616 | 0.0627 | **0.0441** | 0.3534 | 0.6535 | 0.8764 | **0.0047** | **0.0003** | 0.2634 |
| 2011 | 0.9987 | 0.0000 | 1.0000 | 1.0000 | 1.0000 | 0.9923 | 0.8725 | 0.9270 | 0.9798 | 1.0000 | 0.9987 | 0.4481 | 0.2608 | 0.9957 |
| 2012 | 0.9984 | 1.0000 | 0.0000 | 1.0000 | 1.0000 | 0.9999 | 0.9845 | 0.9953 | 0.9987 | 1.0000 | 0.9984 | 0.7980 | 0.6592 | 0.9999 |
| 2013 | 0.9664 | 1.0000 | 1.0000 | 0.0000 | 1.0000 | 0.9912 | 0.8469 | 0.9067 | 0.9790 | 1.0000 | 0.9664 | 0.3754 | 0.1828 | 0.9954 |
| 2014 | 0.9989 | 1.0000 | 1.0000 | 1.0000 | 0.0000 | 0.8091 | 0.4537 | 0.4991 | 0.8204 | 0.9959 | 0.9989 | 0.0948 | **0.0241** | 0.8727 |
| 2015 | 0.1616 | 0.9923 | 0.9999 | 0.9912 | 0.8091 | 0.0000 | 1.0000 | 1.0000 | 1.0000 | 1.0000 | 0.1616 | 0.9813 | 0.9323 | 1.0000 |
| 2016 | 0.0627 | 0.8725 | 0.9845 | 0.8469 | 0.4537 | 1.0000 | 0.0000 | 1.0000 | 1.0000 | 0.9867 | 0.0627 | 1.0000 | 0.9999 | 1.0000 |
| 2017 | **0.0441** | 0.9270 | 0.9953 | 0.9067 | 0.4991 | 1.0000 | 1.0000 | 0.0000 | 1.0000 | 0.9967 | **0.0441** | 0.9994 | 0.9953 | 1.0000 |
| 2018 | 0.3534 | 0.9798 | 0.9987 | 0.9790 | 0.8204 | 1.0000 | 1.0000 | 1.0000 | 0.0000 | 0.9995 | 0.3534 | 0.9998 | 0.9989 | 1.0000 |
| 2019 | 0.6535 | 1.0000 | 1.0000 | 1.0000 | 0.9959 | 1.0000 | 0.9867 | 0.9967 | 0.9995 | 0.0000 | 0.6535 | 0.7358 | 0.5205 | 1.0000 |
| 2020 | 0.8764 | 0.9987 | 0.9984 | 0.9664 | 0.9989 | 0.1616 | 0.0627 | **0.0441** | 0.3534 | 0.6535 | 0.0000 | **0.0047** | **0.0003** | 0.2634 |
| 2021 | **0.0047** | 0.4481 | 0.7980 | 0.3754 | 0.0948 | 0.9813 | 1.0000 | 0.9994 | 0.9998 | 0.7358 | **0.0047** | 0.0000 | 1.0000 | 0.9808 |
| 2022 | **0.0003** | 0.2608 | 0.6592 | 0.1828 | **0.0241** | 0.9323 | 0.9999 | 0.9953 | 0.9989 | 0.5205 | **0.0003** | 1.0000 | 0.0000 | 0.9338 |
| 2023 | 0.2634 | 0.9957 | 0.9999 | 0.9954 | 0.8727 | 1.0000 | 1.0000 | 1.0000 | 1.0000 | 1.0000 | 0.2634 | 0.9808 | 0.9338 | 0.0000 |
| Motive = Trophy hunting | | | | | | | | | | | | | | |
| Year | 2010 | 2011 | 2012 | 2013 | 2014 | 2015 | 2016 | 2017 | 2018 | 2019 | 2020 | 2021 | 2022 | 2023 |
| 2010 | 0.0000 | 0.9998 | 0.9957 | 0.4786 | **0.0324** | **0.0011** | **<0.0001** | **<0.0001** | **<0.0001** | **0.0009** | **<0.0001** | **0.0001** | **<0.0001** | **0.0059** |
| 2011 | 0.9998 | 0.0000 | 1.0000 | 0.9835 | 0.4652 | 0.1075 | **0.0044** | **0.0030** | **0.0017** | 0.0856 | **0.0001** | **0.0228** | **0.0171** | 0.2444 |
| 2012 | 0.9957 | 1.0000 | 0.0000 | 1.0000 | 0.9213 | 0.6384 | 0.1500 | 0.1307 | 0.0843 | 0.5707 | **0.0177** | 0.3327 | 0.3114 | 0.8122 |
| 2013 | 0.4786 | 0.9835 | 1.0000 | 0.0000 | 0.9968 | 0.8707 | 0.1903 | 0.1521 | 0.0921 | 0.8098 | **0.0076** | 0.4938 | 0.4493 | 0.9736 |
| 2014 | **0.0324** | 0.4652 | 0.9213 | 0.9968 | 0.0000 | 1.0000 | 0.9431 | 0.9258 | 0.8125 | 1.0000 | 0.3345 | 0.9978 | 0.9975 | 1.0000 |
| 2015 | **0.0011** | 0.1075 | 0.6384 | 0.8707 | 1.0000 | 0.0000 | 0.9912 | 0.9857 | 0.9245 | 1.0000 | 0.3627 | 1.0000 | 1.0000 | 1.0000 |
| 2016 | **<0.0001** | **0.0044** | 0.1500 | 0.1903 | 0.9431 | 0.9912 | 0.0000 | 1.0000 | 1.0000 | 0.9989 | 0.9918 | 1.0000 | 1.0000 | 0.9532 |
| 2017 | **<0.0001** | **0.0030** | 0.1307 | 0.1521 | 0.9258 | 0.9857 | 1.0000 | 0.0000 | 1.0000 | 0.9981 | 0.9849 | 1.0000 | 1.0000 | 0.9336 |
| 2018 | **<0.0001** | **0.0017** | 0.0843 | 0.0921 | 0.8125 | 0.9245 | 1.0000 | 1.0000 | 0.0000 | 0.9792 | 1.0000 | 0.9993 | 0.9980 | 0.8127 |
| 2019 | **0.0009** | 0.0856 | 0.5707 | 0.8098 | 1.0000 | 1.0000 | 0.9989 | 0.9981 | 0.9792 | 0.0000 | 0.6000 | 1.0000 | 1.0000 | 1.0000 |
| 2020 | **<0.0001** | **0.0001** | **0.0177** | **0.0076** | 0.3345 | 0.3627 | 0.9918 | 0.9849 | 1.0000 | 0.6000 | 0.0000 | 0.8472 | 0.7174 | 0.2482 |
| 2021 | **0.0001** | **0.0228** | 0.3327 | 0.4938 | 0.9978 | 1.0000 | 1.0000 | 1.0000 | 0.9993 | 1.0000 | 0.8472 | 0.0000 | 1.0000 | 0.9993 |
| 2022 | **<0.0001** | **0.0171** | 0.3114 | 0.4493 | 0.9975 | 1.0000 | 1.0000 | 1.0000 | 0.9980 | 1.0000 | 0.7174 | 1.0000 | 0.0000 | 0.9991 |
| 2023 | **0.0059** | 0.2444 | 0.8122 | 0.9736 | 1.0000 | 1.0000 | 0.9532 | 0.9336 | 0.8127 | 1.0000 | 0.2482 | 0.9993 | 0.9991 | 0.0000 |
| Motive = Unknown | | | | | | | | | | | | | | |
| Year | 2010 | 2011 | 2012 | 2013 | 2014 | 2015 | 2016 | 2017 | 2018 | 2019 | 2020 | 2021 | 2022 | 2023 |
| 2010 | 0.0000 | 1.0000 | 1.0000 | 0.6144 | 0.1871 | 0.1616 | 0.9694 | **0.0201** | 0.9989 | **0.0264** | 0.2108 | 0.8564 | 0.8678 | 1.0000 |
| 2011 | 1.0000 | 0.0000 | 1.0000 | 0.6144 | 0.1871 | 0.1616 | 0.9694 | **0.0201** | 0.9989 | **0.0264** | 0.2108 | 0.8564 | 0.8678 | 1.0000 |
| 2012 | 1.0000 | 1.0000 | 0.0000 | 0.6144 | 0.1871 | 0.1616 | 0.9694 | **0.0201** | 0.9989 | **0.0264** | 0.2108 | 0.8564 | 0.8678 | 1.0000 |
| 2013 | 0.6144 | 0.6144 | 0.6144 | 0.0000 | 0.9999 | 1.0000 | 0.9959 | 0.9997 | 0.9817 | 0.9967 | 1.0000 | 0.9999 | 0.9963 | 0.6144 |
| 2014 | 0.1871 | 0.1871 | 0.1871 | 0.9999 | 0.0000 | 1.0000 | 0.7762 | 1.0000 | 0.6588 | 1.0000 | 1.0000 | 0.9173 | 0.7728 | 0.1871 |
| 2015 | 0.1616 | 0.1616 | 0.1616 | 1.0000 | 1.0000 | 0.0000 | 0.9330 | 1.0000 | 0.8407 | 0.9993 | 1.0000 | 0.9925 | 0.9295 | 0.1616 |
| 2016 | 0.9694 | 0.9694 | 0.9694 | 0.9959 | 0.7762 | 0.9330 | 0.0000 | 0.5126 | 1.0000 | 0.4220 | 0.8473 | 1.0000 | 1.0000 | 0.9694 |
| 2017 | **0.0201** | **0.0201** | **0.0201** | 0.9997 | 1.0000 | 1.0000 | 0.5126 | 0.0000 | 0.3661 | 1.0000 | 1.0000 | 0.7772 | 0.4871 | **0.0201** |
| 2018 | 0.9989 | 0.9989 | 0.9989 | 0.9817 | 0.6588 | 0.8407 | 1.0000 | 0.3661 | 0.0000 | 0.3008 | 0.7382 | 1.0000 | 1.0000 | 0.9989 |
| 2019 | **0.0264** | **0.0264** | **0.0264** | 0.9967 | 1.0000 | 0.9993 | 0.4220 | 1.0000 | 0.3008 | 0.0000 | 1.0000 | 0.6649 | 0.4037 | **0.0264** |
| 2020 | 0.2108 | 0.2108 | 0.2108 | 1.0000 | 1.0000 | 1.0000 | 0.8473 | 1.0000 | 0.7382 | 1.0000 | 0.0000 | 0.9577 | 0.8446 | 0.2108 |
| 2021 | 0.8564 | 0.8564 | 0.8564 | 0.9999 | 0.9173 | 0.9925 | 1.0000 | 0.7772 | 1.0000 | 0.6649 | 0.9577 | 0.0000 | 1.0000 | 0.8564 |
| 2022 | 0.8678 | 0.8678 | 0.8678 | 0.9963 | 0.7728 | 0.9295 | 1.0000 | 0.4871 | 1.0000 | 0.4037 | 0.8446 | 1.0000 | 0.0000 | 0.8678 |
| 2023 | 1.0000 | 1.0000 | 1.0000 | 0.6144 | 0.1871 | 0.1616 | 0.9694 | **0.0201** | 0.9989 | **0.0264** | 0.2108 | 0.8564 | 0.8678 | 0.0000 |

| Method = Gin trap | | | | | | | | | | | | | | |
| --- | --- | --- | --- | --- | --- | --- | --- | --- | --- | --- | --- | --- | --- | --- |
| Year | 2010 | 2011 | 2012 | 2013 | 2014 | 2015 | 2016 | 2017 | 2018 | 2019 | 2020 | 2021 | 2022 | 2023 |
| 2010 | 1.0000 | 0.6247 | 0.6245 | 0.9989 | 0.9650 | 0.8597 | 0.2599 | 0.9990 | 0.5950 | 0.9989 | 0.9663 | 0.9989 | 0.6890 | **0.0126** |
| 2011 | 0.6247 | 1.0000 | 0.5470 | 0.9989 | 0.9650 | 0.8597 | 0.2599 | 0.9990 | 0.5950 | 0.9989 | 0.9663 | 0.9989 | 0.6890 | **0.0126** |
| 2012 | 0.6245 | 0.5470 | 1.0000 | 0.9989 | 0.9650 | 0.8597 | 0.2599 | 0.9990 | 0.5950 | 0.9989 | 0.9663 | 0.9989 | 0.6890 | **0.0126** |
| 2013 | 0.9989 | 0.9989 | 0.9989 | 1.0000 | 1.0000 | 1.0000 | 0.8260 | 1.0000 | 0.9504 | 1.0000 | 1.0000 | 1.0000 | 0.9999 | 0.1486 |
| 2014 | 0.9650 | 0.9650 | 0.9650 | 1.0000 | 1.0000 | 1.0000 | 0.9957 | 0.9994 | 0.9994 | 0.9999 | 1.0000 | 0.9998 | 1.0000 | 0.5733 |
| 2015 | 0.8597 | 0.8597 | 0.8597 | 1.0000 | 1.0000 | 1.0000 | 0.9845 | 0.9979 | 0.9978 | 0.9997 | 1.0000 | 0.9994 | 1.0000 | 0.4047 |
| 2016 | 0.2599 | 0.2599 | 0.2599 | 0.8260 | 0.9957 | 0.9845 | 1.0000 | 0.5846 | 1.0000 | 0.6937 | 0.9886 | 0.6579 | 0.9926 | 0.9973 |
| 2017 | 0.9990 | 0.9990 | 0.9990 | 1.0000 | 0.9994 | 0.9979 | 0.5846 | 1.0000 | 0.8452 | 1.0000 | 0.9997 | 1.0000 | 0.9875 | 0.0539 |
| 2018 | 0.5950 | 0.5950 | 0.5950 | 0.9504 | 0.9994 | 0.9978 | 1.0000 | 0.8452 | 1.0000 | 0.8982 | 0.9982 | 0.8819 | 0.9992 | 0.9971 |
| 2019 | 0.9989 | 0.9989 | 0.9989 | 1.0000 | 0.9999 | 0.9997 | 0.6937 | 1.0000 | 0.8982 | 1.0000 | 1.0000 | 1.0000 | 0.9975 | 0.0834 |
| 2020 | 0.9663 | 0.9663 | 0.9663 | 1.0000 | 1.0000 | 1.0000 | 0.9886 | 0.9997 | 0.9982 | 1.0000 | 1.0000 | 0.9999 | 1.0000 | 0.4666 |
| 2021 | 0.9989 | 0.9989 | 0.9989 | 1.0000 | 0.9998 | 0.9994 | 0.6579 | 1.0000 | 0.8819 | 1.0000 | 0.9999 | 1.0000 | 0.9956 | 0.0721 |
| 2022 | 0.6890 | 0.6890 | 0.6890 | 0.9999 | 1.0000 | 1.0000 | 0.9926 | 0.9875 | 0.9992 | 0.9975 | 1.0000 | 0.9956 | 1.0000 | 0.4650 |
| 2023 | **0.0126** | **0.0126** | **0.0126** | 0.1486 | 0.5733 | 0.4047 | 0.9973 | 0.0539 | 0.9971 | 0.0834 | 0.4666 | 0.0721 | 0.4650 | 1.0000 |
| Method = Poisoned | | | | | | | | | | | | | | |
| Year | 2010 | 2011 | 2012 | 2013 | 2014 | 2015 | 2016 | 2017 | 2018 | 2019 | 2020 | 2021 | 2022 | 2023 |
| 2010 | 1.0000 | 0.5472 | 0.5471 | 0.9657 | 0.6055 | 0.4671 | 0.6534 | 0.1748 | 0.9641 | 0.9687 | 0.2148 | **0.0053** | **0.0276** | 0.5733 |
| 2011 | 0.5472 | 1.0000 | 0.2919 | 0.9657 | 0.6055 | 0.4671 | 0.6534 | 0.1748 | 0.9641 | 0.9687 | 0.2148 | **0.0053** | **0.0276** | 0.3035 |
| 2012 | 0.5471 | 0.2919 | 1.0000 | 0.9657 | 0.6055 | 0.4671 | 0.6534 | 0.1748 | 0.9641 | 0.9687 | 0.2148 | **0.0053** | **0.0276** | 0.2960 |
| 2013 | 0.9657 | 0.9657 | 0.9657 | 1.0000 | 0.9995 | 1.0000 | 1.0000 | 0.9957 | 1.0000 | 1.0000 | 0.9528 | 0.3783 | 0.8899 | 0.9657 |
| 2014 | 0.6055 | 0.6055 | 0.6055 | 0.9995 | 1.0000 | 1.0000 | 1.0000 | 1.0000 | 0.9999 | 0.9934 | 1.0000 | 0.9773 | 1.0000 | 0.6055 |
| 2015 | 0.4671 | 0.4671 | 0.4671 | 1.0000 | 1.0000 | 1.0000 | 1.0000 | 1.0000 | 1.0000 | 0.9990 | 0.9990 | 0.7523 | 0.9974 | 0.4671 |
| 2016 | 0.6534 | 0.6534 | 0.6534 | 1.0000 | 1.0000 | 1.0000 | 1.0000 | 1.0000 | 1.0000 | 0.9998 | 0.9983 | 0.7350 | 0.9960 | 0.6534 |
| 2017 | 0.1748 | 0.1748 | 0.1748 | 0.9957 | 1.0000 | 1.0000 | 1.0000 | 1.0000 | 0.9989 | 0.9555 | 1.0000 | 0.9582 | 1.0000 | 0.1748 |
| 2018 | 0.9641 | 0.9641 | 0.9641 | 1.0000 | 0.9999 | 1.0000 | 1.0000 | 0.9989 | 1.0000 | 1.0000 | 0.9767 | 0.4930 | 0.9470 | 0.9641 |
| 2019 | 0.9687 | 0.9687 | 0.9687 | 1.0000 | 0.9934 | 0.9990 | 0.9998 | 0.9555 | 1.0000 | 1.0000 | 0.8461 | 0.1796 | 0.6563 | 0.9687 |
| 2020 | 0.2148 | 0.2148 | 0.2148 | 0.9528 | 1.0000 | 0.9990 | 0.9983 | 1.0000 | 0.9767 | 0.8461 | 1.0000 | 0.9999 | 1.0000 | 0.2148 |
| 2021 | **0.0053** | **0.0053** | **0.0053** | 0.3783 | 0.9773 | 0.7523 | 0.7350 | 0.9582 | 0.4930 | 0.1796 | 0.9999 | 1.0000 | 0.9990 | **0.0053** |
| 2022 | **0.0276** | **0.0276** | **0.0276** | 0.8899 | 1.0000 | 0.9974 | 0.9960 | 1.0000 | 0.9470 | 0.6563 | 1.0000 | 0.9990 | 1.0000 | **0.0276** |
| 2023 | 0.5733 | 0.3035 | 0.2960 | 0.9657 | 0.6055 | 0.4671 | 0.6534 | 0.1748 | 0.9641 | 0.9687 | 0.2148 | **0.0053** | **0.0276** | 1.0000 |
| Method = Shot | | | | | | | | | | | | | | |
| Year | 2010 | 2011 | 2012 | 2013 | 2014 | 2015 | 2016 | 2017 | 2018 | 2019 | 2020 | 2021 | 2022 | 2023 |
| 2010 | 1.0000 | 0.9998 | 1.0000 | 0.4802 | **0.0347** | **0.0013** | **0.0003** | **<0.0001** | **<0.0001** | **0.0035** | **<0.0001** | **0.0004** | **0.0407** | **0.0067** |
| 2011 | 0.9998 | 1.0000 | 1.0000 | 0.9831 | 0.4671 | 0.1113 | **0.0398** | **0.0035** | **0.0020** | 0.1747 | **0.0001** | 0.0558 | 0.6197 | 0.2483 |
| 2012 | 1.0000 | 1.0000 | 1.0000 | 0.9733 | 0.4590 | 0.1273 | 0.0503 | **0.0061** | **0.0036** | 0.1885 | **0.0003** | 0.0683 | 0.6106 | 0.2599 |
| 2013 | 0.4802 | 0.9831 | 0.9733 | 1.0000 | 0.9967 | 0.8694 | 0.6092 | 0.1562 | 0.0957 | 0.9333 | **0.0086** | 0.7025 | 1.0000 | 0.9729 |
| 2014 | **0.0347** | 0.4671 | 0.4590 | 0.9967 | 1.0000 | 1.0000 | 0.9994 | 0.9246 | 0.8113 | 1.0000 | 0.3378 | 0.9999 | 1.0000 | 1.0000 |
| 2015 | **0.0013** | 0.1113 | 0.1273 | 0.8694 | 1.0000 | 1.0000 | 1.0000 | 0.9853 | 0.9234 | 1.0000 | 0.3658 | 1.0000 | 0.9928 | 1.0000 |
| 2016 | **0.0003** | **0.0398** | 0.0503 | 0.6092 | 0.9994 | 1.0000 | 1.0000 | 1.0000 | 0.9977 | 1.0000 | 0.8022 | 1.0000 | 0.8874 | 0.9999 |
| 2017 | **<0.0001** | **0.0035** | **0.0061** | 0.1562 | 0.9246 | 0.9853 | 1.0000 | 1.0000 | 1.0000 | 0.9793 | 0.9845 | 0.9995 | 0.2869 | 0.9326 |
| 2018 | **<0.0001** | **0.0020** | **0.0036** | 0.0957 | 0.8113 | 0.9234 | 0.9977 | 1.0000 | 1.0000 | 0.9102 | 1.0000 | 0.9901 | 0.1777 | 0.8115 |
| 2019 | **0.0035** | 0.1747 | 0.1885 | 0.9333 | 1.0000 | 1.0000 | 1.0000 | 0.9793 | 0.9102 | 1.0000 | 0.3923 | 1.0000 | 0.9985 | 1.0000 |
| 2020 | **<0.0001** | **0.0001** | **0.0003** | **0.0086** | 0.3378 | 0.3658 | 0.8022 | 0.9845 | 1.0000 | 0.3923 | 1.0000 | 0.6636 | **0.0076** | 0.2521 |
| 2021 | **0.0004** | 0.0558 | 0.0683 | 0.7025 | 0.9999 | 1.0000 | 1.0000 | 0.9995 | 0.9901 | 1.0000 | 0.6636 | 1.0000 | 0.9423 | 1.0000 |
| 2022 | **0.0407** | 0.6197 | 0.6106 | 1.0000 | 1.0000 | 0.9928 | 0.8874 | 0.2869 | 0.1777 | 0.9985 | **0.0076** | 0.9423 | 1.0000 | 0.9999 |
| 2023 | **0.0067** | 0.2483 | 0.2599 | 0.9729 | 1.0000 | 1.0000 | 0.9999 | 0.9326 | 0.8115 | 1.0000 | 0.2521 | 1.0000 | 0.9999 | 1.0000 |
| Method = Snare | | | | | | | | | | | | | | |
| Year | 2010 | 2011 | 2012 | 2013 | 2014 | 2015 | 2016 | 2017 | 2018 | 2019 | 2020 | 2021 | 2022 | 2023 |
| 2010 | 1.0000 | 0.7622 | 0.9393 | 0.9657 | 0.9988 | 0.8598 | 0.0659 | 0.6791 | **0.0068** | 0.2599 | 0.1010 | 0.4511 | 0.6891 | 0.9690 |
| 2011 | 0.7622 | 1.0000 | 1.0000 | 0.9976 | 0.9650 | 0.9962 | 1.0000 | 0.9994 | 0.9873 | 1.0000 | 1.0000 | 1.0000 | 0.9981 | 0.9862 |
| 2012 | 0.9393 | 1.0000 | 1.0000 | 0.9999 | 0.9957 | 0.9998 | 1.0000 | 1.0000 | 0.9791 | 1.0000 | 1.0000 | 1.0000 | 0.9999 | 0.9988 |
| 2013 | 0.9657 | 0.9976 | 0.9999 | 1.0000 | 1.0000 | 1.0000 | 0.8457 | 1.0000 | 0.2099 | 0.9928 | 0.8265 | 0.9999 | 1.0000 | 1.0000 |
| 2014 | 0.9988 | 0.9650 | 0.9957 | 1.0000 | 1.0000 | 1.0000 | 0.4558 | 0.9996 | 0.0606 | 0.8508 | 0.4739 | 0.9744 | 0.9999 | 1.0000 |
| 2015 | 0.8598 | 0.9962 | 0.9998 | 1.0000 | 1.0000 | 1.0000 | 0.7693 | 1.0000 | 0.1543 | 0.9845 | 0.7585 | 0.9997 | 1.0000 | 1.0000 |
| 2016 | 0.0659 | 1.0000 | 1.0000 | 0.8457 | 0.4558 | 0.7693 | 1.0000 | 0.9012 | 0.9933 | 1.0000 | 1.0000 | 0.9976 | 0.8240 | 0.6038 |
| 2017 | 0.6791 | 0.9994 | 1.0000 | 1.0000 | 0.9996 | 1.0000 | 0.9012 | 1.0000 | 0.2489 | 0.9981 | 0.8839 | 1.0000 | 1.0000 | 1.0000 |
| 2018 | **0.0068** | 0.9873 | 0.9791 | 0.2099 | 0.0606 | 0.1543 | 0.9933 | 0.2489 | 1.0000 | 0.8688 | 0.9988 | 0.6007 | 0.1823 | 0.0943 |
| 2019 | 0.2599 | 1.0000 | 1.0000 | 0.9928 | 0.8508 | 0.9845 | 1.0000 | 0.9981 | 0.8688 | 1.0000 | 1.0000 | 1.0000 | 0.9926 | 0.9375 |
| 2020 | 0.1010 | 1.0000 | 1.0000 | 0.8265 | 0.4739 | 0.7585 | 1.0000 | 0.8839 | 0.9988 | 1.0000 | 1.0000 | 0.9947 | 0.8103 | 0.6088 |
| 2021 | 0.4511 | 1.0000 | 1.0000 | 0.9999 | 0.9744 | 0.9997 | 0.9976 | 1.0000 | 0.6007 | 1.0000 | 0.9947 | 1.0000 | 0.9999 | 0.9953 |
| 2022 | 0.6891 | 0.9981 | 0.9999 | 1.0000 | 0.9999 | 1.0000 | 0.8240 | 1.0000 | 0.1823 | 0.9926 | 0.8103 | 0.9999 | 1.0000 | 1.0000 |
| 2023 | 0.9690 | 0.9862 | 0.9988 | 1.0000 | 1.0000 | 1.0000 | 0.6038 | 1.0000 | 0.0943 | 0.9375 | 0.6088 | 0.9953 | 1.0000 | 1.0000 |
| Method = Unknown | | | | | | | | | | | | | | |
| Year | 2010 | 2011 | 2012 | 2013 | 2014 | 2015 | 2016 | 2017 | 2018 | 2019 | 2020 | 2021 | 2022 | 2023 |
| 2010 | 1.0000 | 0.9984 | 0.9984 | 0.9997 | 0.9896 | 0.7912 | 1.0000 | 0.3252 | 1.0000 | 0.8443 | 0.9966 | 1.0000 | 1.0000 | 0.9986 |
| 2011 | 0.9984 | 1.0000 | 0.9961 | 0.3832 | 0.1911 | **0.0030** | 0.8514 | **<0.0001** | 0.9641 | **0.0110** | 0.2148 | 0.4511 | 0.6891 | 0.1441 |
| 2012 | 0.9984 | 0.9961 | 1.0000 | 0.3832 | 0.1911 | **0.0030** | 0.8514 | **<0.0001** | 0.9641 | **0.0110** | 0.2148 | 0.4511 | 0.6891 | 0.1441 |
| 2013 | 0.9997 | 0.3832 | 0.3832 | 1.0000 | 1.0000 | 0.9980 | 0.9965 | 0.8500 | 0.9967 | 0.9991 | 1.0000 | 1.0000 | 0.9916 | 1.0000 |
| 2014 | 0.9896 | 0.1911 | 0.1911 | 1.0000 | 1.0000 | 1.0000 | 0.9436 | 0.9904 | 0.9489 | 1.0000 | 1.0000 | 0.9988 | 0.9056 | 1.0000 |
| 2015 | 0.7912 | **0.0030** | **0.0030** | 0.9980 | 1.0000 | 1.0000 | 0.4112 | 0.9999 | 0.4673 | 1.0000 | 0.9999 | 0.8544 | 0.2877 | 0.9985 |
| 2016 | 1.0000 | 0.8514 | 0.8514 | 0.9965 | 0.9436 | 0.4112 | 1.0000 | 0.0564 | 1.0000 | 0.5337 | 0.9740 | 1.0000 | 1.0000 | 0.9829 |
| 2017 | 0.3252 | **<0.0001** | **<0.0001** | 0.8500 | 0.9904 | 0.9999 | 0.0564 | 1.0000 | 0.0802 | 0.9999 | 0.9533 | 0.3042 | **0.0272** | 0.8388 |
| 2018 | 1.0000 | 0.9641 | 0.9641 | 0.9967 | 0.9489 | 0.4673 | 1.0000 | 0.0802 | 1.0000 | 0.5790 | 0.9767 | 1.0000 | 1.0000 | 0.9854 |
| 2019 | 0.8443 | **0.0110** | **0.0110** | 0.9991 | 1.0000 | 1.0000 | 0.5337 | 0.9999 | 0.5790 | 1.0000 | 1.0000 | 0.9089 | 0.4135 | 0.9993 |
| 2020 | 0.9966 | 0.2148 | 0.2148 | 1.0000 | 1.0000 | 0.9999 | 0.9740 | 0.9533 | 0.9767 | 1.0000 | 1.0000 | 0.9998 | 0.9499 | 1.0000 |
| 2021 | 1.0000 | 0.4511 | 0.4511 | 1.0000 | 0.9988 | 0.8544 | 1.0000 | 0.3042 | 1.0000 | 0.9089 | 0.9998 | 1.0000 | 0.9999 | 1.0000 |
| 2022 | 1.0000 | 0.6891 | 0.6891 | 0.9916 | 0.9056 | 0.2877 | 1.0000 | **0.0272** | 1.0000 | 0.4135 | 0.9499 | 0.9999 | 1.0000 | 0.9611 |
| 2023 | 0.9986 | 0.1441 | 0.1441 | 1.0000 | 1.0000 | 0.9985 | 0.9829 | 0.8388 | 0.9854 | 0.9993 | 1.0000 | 1.0000 | 0.9611 | 1.0000 |
| Method = Vehicular collision | | | | | | | | | | | | | | |
| Year | 2010 | 2011 | 2012 | 2013 | 2014 | 2015 | 2016 | 2017 | 2018 | 2019 | 2020 | 2021 | 2022 | 2023 |
| 2010 | 1.0000 | 1.0000 | 1.0000 | 1.0000 | 1.0000 | 1.0000 | 1.0000 | 0.9990 | 1.0000 | 1.0000 | 1.0000 | 1.0000 | 1.0000 | 1.0000 |
| 2011 | 1.0000 | 1.0000 | 1.0000 | 1.0000 | 1.0000 | 1.0000 | 1.0000 | 0.9990 | 1.0000 | 1.0000 | 1.0000 | 1.0000 | 1.0000 | 1.0000 |
| 2012 | 1.0000 | 1.0000 | 1.0000 | 1.0000 | 1.0000 | 1.0000 | 1.0000 | 0.9990 | 1.0000 | 1.0000 | 1.0000 | 1.0000 | 1.0000 | 1.0000 |
| 2013 | 1.0000 | 1.0000 | 1.0000 | 1.0000 | 1.0000 | 1.0000 | 1.0000 | 0.9990 | 1.0000 | 1.0000 | 1.0000 | 1.0000 | 1.0000 | 1.0000 |
| 2014 | 1.0000 | 1.0000 | 1.0000 | 1.0000 | 1.0000 | 1.0000 | 1.0000 | 0.9990 | 1.0000 | 1.0000 | 1.0000 | 1.0000 | 1.0000 | 1.0000 |
| 2015 | 1.0000 | 1.0000 | 1.0000 | 1.0000 | 1.0000 | 1.0000 | 1.0000 | 0.9990 | 1.0000 | 1.0000 | 1.0000 | 1.0000 | 1.0000 | 1.0000 |
| 2016 | 1.0000 | 1.0000 | 1.0000 | 1.0000 | 1.0000 | 1.0000 | 1.0000 | 0.9990 | 1.0000 | 1.0000 | 1.0000 | 1.0000 | 1.0000 | 1.0000 |
| 2017 | 0.9990 | 0.9990 | 0.9990 | 0.9990 | 0.9990 | 0.9990 | 0.9990 | 1.0000 | 0.9990 | 0.9990 | 0.9990 | 0.9990 | 0.9990 | 0.9990 |
| 2018 | 1.0000 | 1.0000 | 1.0000 | 1.0000 | 1.0000 | 1.0000 | 1.0000 | 0.9990 | 1.0000 | 1.0000 | 1.0000 | 1.0000 | 1.0000 | 1.0000 |
| 2019 | 1.0000 | 1.0000 | 1.0000 | 1.0000 | 1.0000 | 1.0000 | 1.0000 | 0.9990 | 1.0000 | 1.0000 | 1.0000 | 1.0000 | 1.0000 | 1.0000 |
| 2020 | 1.0000 | 1.0000 | 1.0000 | 1.0000 | 1.0000 | 1.0000 | 1.0000 | 0.9990 | 1.0000 | 1.0000 | 1.0000 | 1.0000 | 1.0000 | 1.0000 |
| 2021 | 1.0000 | 1.0000 | 1.0000 | 1.0000 | 1.0000 | 1.0000 | 1.0000 | 0.9990 | 1.0000 | 1.0000 | 1.0000 | 1.0000 | 1.0000 | 1.0000 |
| 2022 | 1.0000 | 1.0000 | 1.0000 | 1.0000 | 1.0000 | 1.0000 | 1.0000 | 0.9990 | 1.0000 | 1.0000 | 1.0000 | 1.0000 | 1.0000 | 1.0000 |
| 2023 | 1.0000 | 1.0000 | 1.0000 | 1.0000 | 1.0000 | 1.0000 | 1.0000 | 0.9990 | 1.0000 | 1.0000 | 1.0000 | 1.0000 | 1.0000 | 1.0000 |

**Table S2 (cont.). Tukey post-hoc test results derived from the multinomial linear regression models.**

(B) Spatial differences by motive and method. Presented are Tukey post-hoc tests with significant pairwise comparisons (*P* < 0.05) highlighted in bold.

| Motive = Accidental | | | | | |
| --- | --- | --- | --- | --- | --- |
| Contrast | Estimate | SE | df | t ratio | p value |
| Central-Northern | -0.0049 | 0.0049 | 24 | -1.002 | 0.7495 |
| Central-Outside | 0.0000 | 0.0000 | 24 | -0.648 | 0.9153 |
| Central-Southern | 0.0000 | 0.00000073 | 24 | -0.007 | 1.0000 |
| Northern-Outside | 0.0049 | 0.0049 | 24 | 1.002 | 0.7495 |
| Northern-Southern | 0.0049 | 0.0049 | 24 | 1.002 | 0.7495 |
| Outside-Southern | 0.0000 | 0.00000073 | 24 | -0.006 | 1.0000 |
| Motive = Bushmeat bycatch | | | | | |
| Contrast | Estimate | SE | df | t ratio | p value |
| **Central-Northern** | **0.7645** | **0.0510** | **24** | **15.001** | **<0.0001** |
| **Central-Outside** | **0.7281** | **0.0762** | **24** | **9.551** | **<0.0001** |
| **Central-Southern** | **0.7429** | **0.0713** | **24** | **10.424** | **<0.0001** |
| Northern-Outside | -0.0363 | 0.0635 | 24 | -0.572 | 0.9394 |
| Northern-Southern | -0.0216 | 0.0575 | 24 | -0.376 | 0.9815 |
| Outside-Southern | 0.0148 | 0.0807 | 24 | 0.183 | 0.9978 |
| Motive = Damage causing animal | | | | | |
| Contrast | Estimate | SE | df | t ratio | p value |
| Central-Northern | -0.0098 | 0.0069 | 24 | -1.421 | 0.4989 |
| Central-Outside | -0.0323 | 0.0317 | 24 | -1.017 | 0.7415 |
| Central-Southern | 0.0000 | 0.00000242 | 24 | -0.006 | 1.0000 |
| Northern-Outside | -0.0225 | 0.0325 | 24 | -0.693 | 0.8987 |
| Northern-Southern | 0.0098 | 0.0069 | 24 | 1.421 | 0.4989 |
| Outside-Southern | 0.0323 | 0.0317 | 24 | 1.017 | 0.7415 |
| Motive = Retaliation | | | | | |
| Contrast | Estimate | SE | df | t ratio | p value |
| **Central-Northern** | **-0.1024** | **0.0212** | **24** | **-4.837** | **0.0003** |
| Central-Outside | -0.1290 | 0.0602 | 24 | -2.143 | 0.1684 |
| **Central-Southern** | **-0.2857** | **0.0764** | **24** | **-3.742** | **0.0052** |
| Northern-Outside | -0.0266 | 0.0638 | 24 | -0.417 | 0.9751 |
| Northern-Southern | -0.1833 | 0.0792 | 24 | -2.313 | 0.1231 |
| Outside-Southern | -0.1567 | 0.0972 | 24 | -1.611 | 0.3914 |
| Motive = Targeted poaching | | | | | |
| Contrast | Estimate | SE | df | t ratio | p value |
| Central-Northern | -0.0880 | 0.0497 | 24 | -1.795 | 0.3123 |
| Central-Outside | 0.0103 | 0.0673 | 24 | 0.154 | 0.9987 |
| **Central-Southern** | **-0.4929** | **0.0925** | **24** | **-5.325** | **0.0001** |
| Northern-Outside | 0.0983 | 0.0599 | 24 | 1.642 | 0.3754 |
| **Northern-Southern** | **-0.4049** | **0.0873** | **24** | **-4.637** | **0.0006** |
| **Outside-Southern** | **-0.5032** | **0.0984** | **24** | **-5.115** | **0.0002** |
| Motive = Trophy hunting | | | | | |
| Contrast | Estimate | SE | df | t ratio | p value |
| **Central-Northern** | **-0.3106** | **0.0415** | **24** | **-7.491** | **<0.0001** |
| **Central-Outside** | **-0.5772** | **0.0909** | **24** | **-6.347** | **<0.0001** |
| Central-Southern | 0.0357 | 0.0248 | 24 | 1.44 | 0.4877 |
| **Northern-Outside** | **-0.2665** | **0.0936** | **24** | **-2.848** | **0.0412** |
| **Northern-Southern** | **0.3463** | **0.0332** | **24** | **10.422** | **<0.0001** |
| **Outside-Southern** | **0.6129** | **0.0875** | **24** | **7.006** | **<0.0001** |
| Motive = Unknown | | | | | |
| Contrast | Estimate | SE | df | t ratio | p value |
| **Central-Northern** | **-0.2488** | **0.0302** | **24** | **-8.239** | **<0.0001** |
| Central-Outside | 0.0000 | 0.0000 | 24 | -0.195 | 0.9973 |
| Central-Southern | 0.0000 | 0.00000093 | 24 | -0.006 | 1.0000 |
| **Northern-Outside** | **0.2488** | **0.0302** | **24** | **8.239** | **<0.0001** |
| **Northern-Southern** | **0.2488** | **0.0302** | **24** | **8.239** | **<0.0001** |
| Outside-Southern | 0.0000 | 0.00000093 | 24 | -0.006 | 1.0000 |

Tukey post-hoc tests for each method of lion mortality compared by region.

| Method = Gin trap | | | | | |
| --- | --- | --- | --- | --- | --- |
| Contrast | Estimate | SE | df | t ratio | p value |
| **Central-Northern** | **0.4526** | **0.0675** | **20** | **6.857** | **<0.0001** |
| **Central-Outside** | **0.3854** | **0.0853** | **20** | **4.517** | **0.0011** |
| **Central-Southern** | **0.4536** | **0.0725** | **20** | **6.359** | **<0.0001** |
| Northern-Outside | -0.0773 | 0.0540 | 20 | -1.432 | 0.4953 |
| Northern-Southern | -0.0091 | 0.0298 | 20 | -0.304 | 0.9899 |
| Outside-Southern | 0.0682 | 0.0601 | 20 | 1.135 | 0.6730 |
| Method = Poisoned | | | | | |
| Contrast | Estimate | SE | df | t ratio | p value |
| **Central-Northern** | **-0.1561** | **0.0254** | **20** | **-6.158** | **<0.0001** |
| Central-Outside | -0.0968 | 0.0531 | 20 | -1.823 | 0.2924 |
| **Central-Southern** | **-0.5143** | **0.0845** | **20** | **-6.088** | **<0.0001** |
| Northern-Outside | 0.0593 | 0.0588 | 20 | 1.008 | 0.7466 |
| **Northern-Southern** | **-0.3582** | **0.0882** | **20** | **-4.061** | **0.0031** |
| **Outside-Southern** | **-0.4175** | **0.0998** | **20** | **-4.184** | **0.0024** |
| Method = Shot | | | | | |
| Contrast | Estimate | SE | df | t ratio | p value |
| **Central-Northern** | **-0.3399** | **0.0419** | **20** | **-8.103** | **<0.0001** |
| **Central-Outside** | **-0.7385** | **0.0791** | **20** | **-9.337** | **<0.0001** |
| Central-Southern | -0.0214 | 0.0464 | 20 | -0.461 | 0.9666 |
| **Northern-Outside** | **-0.3986** | **0.0824** | **20** | **-4.839** | **0.0005** |
| **Northern-Southern** | **0.3185** | **0.0518** | **20** | **6.148** | **<0.0001** |
| **Outside-Southern** | **0.7171** | **0.0847** | **20** | **8.463** | **<0.0001** |
| Method = Snared | | | | | |
| Contrast | Estimate | SE | df | t ratio | p value |
| **Central-Northern** | **0.3083** | **0.0691** | **20** | **4.463** | **0.0013** |
| **Central-Outside** | **0.3785** | **0.0730** | **20** | **5.184** | **0.0002** |
| Central-Southern | 0.0964 | 0.1024 | 20 | 0.942 | 0.7829 |
| Northern-Outside | 0.0702 | 0.0382 | 20 | 1.839 | 0.2850 |
| Northern-Southern | -0.2119 | 0.0813 | 20 | -2.607 | 0.0736 |
| **Outside-Southern** | **-0.2820** | **0.0846** | **20** | **-3.332** | **0.0161** |
| Method = Vehicular collision | | | | | |
| Contrast | Estimate | SE | df | t ratio | p value |
| Central-Northern | -0.0049 | 0.0049 | 20 | -1.003 | 0.7497 |
| Central-Outside | 0.0000 | 0.0000 | 20 | 1.716 | 0.3419 |
| Central-Southern | 0.0000 | 0.0000 | 20 | 1.194 | 0.6376 |
| Northern-Outside | 0.0049 | 0.0049 | 20 | 1.003 | 0.7497 |
| Northern-Southern | 0.0049 | 0.0049 | 20 | 1.003 | 0.7497 |
| Outside-Southern | 0.0000 | 0.0000 | 20 | -0.682 | 0.9027 |
| Central-Northern | -0.0049 | 0.0049 | 20 | -1.003 | 0.7497 |
| Method = Unknown | | | | | |
| Contrast | Estimate | SE | df | t ratio | p value |
| **Central-Northern** | **-0.2700** | **0.0478** | **20** | **-5.654** | **0.0001** |
| Central-Outside | 0.0714 | 0.0344 | 20 | 2.075 | 0.1951 |
| Central-Southern | -0.0143 | 0.0585 | 20 | -0.244 | 0.9947 |
| **Northern-Outside** | **0.3415** | **0.0331** | **20** | **10.31** | **<0.0001** |
| **Northern-Southern** | **0.2558** | **0.0578** | **20** | **4.428** | **0.0014** |
| Outside-Southern | -0.0857 | 0.0473 | 20 | -1.811 | 0.2973 |
| **Central-Northern** | **-0.2700** | **0.0478** | **20** | **-5.654** | **0.0001** |

**Table S2. Details of estimated detection rates, anthropogenic mortality rates (AMR) and recovery targets for lion populations within Mozambique.** Included are current AMRs, trends in AMRs, and targets for AMR reduction needed to promote lion recovery. Effects of interventions on AMRs are also provided to highlight the importance of monitoring and veterinary capacity.

| **Lion population** | **Detection rate** | **Current AMR** | | **Trend in AMR** | | **AMR reduction targets for population recovery** | |
| --- | --- | --- | --- | --- | --- | --- | --- |
|  |  | **No intervention** | **With current intervention** | **No intervention** | **With current intervention** | **No intervention** | **With current intervention** |
| Coutadas 9/13 | 100% | 8.85% | 6.67% | 0.39% | 0.21% | 5–10% | 5% |
| Gorongosa NP | 100% | 1.43% | 1.08% | -0.03% | -0.02% | 0% | 0% |
| Limpopo NP | 40% (-K)  20% (+K) | 7.93% | 7.93% | -0.11% | -0.11% | 15–20% (-K)  20–40% (+K) | 15–20% (-K)  20–40% (+K) |
| Niassa SR | 20% | 2.71% | 2.6% | 0.11% | 0.10% | 10–15% | 10–15% |
| Zambezi Delta | 100% | 8.26% | 6.29% | 0.79% | 0.21% | 5–10% | 5% |

**References**

Bauer H, Van Der Merwe S. Inventory of free-ranging lions (*Panthera leo*) in Africa. Oryx. 2004;38: 26-31.

Begg CM, Miller JRB, Begg KS. Effective implementation of age restrictions increases selectivity of sport hunting of the African lion. Journal of Applied Ecology. 2018;55: 139–146.

Bouley P, Poulos M, Branco R, Carter NH. Post-war recovery of the African lion in response to large-scale ecosystem restoration. Biological Conservation. 2018;227: 233–242.

Briers-Louw WD, Kendon TA, Rogan MS, Naude VN, Leslie AJ, Gaynor D. Anthropogenic pressure limits the recovery of a post-war leopard population in central Mozambique. Conservation Science and Practice. 2024; e13122.

Chardonnet P. Conservation of the African lion: Contribution to a status survey. International Foundation for the Conservation of Wildlife, France and Conservation Force, USA; 2002. Available: <http://conservationforce.org/pdf/conservationoftheafricanlion.pdf>

﻿Chardonnet P, Mesochina P, Renaud PC, Bento C, Conjo D, Fusari A. et al. Conservation status of the lion (*Panthera leo* Linnaeus 1758) in Mozambique. Maputo; 2009. [http://www.shakariconnection.com/support- files/mozambique-lion-final-report.pdf](http://www.shakariconnection.com/support-%20files/mozambique-lion-final-report.pdf).

Everatt KT, Kokes R, Lopez Pereira C. Evidence of a further emerging threat to lion conservation; targeted poaching for body parts. Biodiversity and Conservation. 2019;28: 4099–4114.

﻿Hanks J. The role of Transfrontier Conservation Areas in southern Africa in the conservation of mammalian biodiversity. In: Entwistle A, Dunstone N, editors. Priorities for the Conservation of Mammalian Diversity. Has the Panda had its day? Cambridge: Cambridge University Press. pp. 239–256; 2000.

﻿Hatton J, Couto M, Oglethorpe J. Biodiversity and war: A case study of Mozambique. Unpublished report. Biodiversity Support Program, Washington, USA; 2001. <https://biblioteca.biofund.org.mz/wp-content/uploads/2018/12/1544786392-F2318.337-Hatton%20Et%20Al%202001%20Mozambique%20Biodiversity%20War.Pdf>

Jacobson AP, Cattau ME, Riggio JS, Petracca LS, Fedak DA. ﻿Distribution and abundance of lions in northwest Tete Province, Mozambique. Tropical Conservation Science. 2013;6: 87–107.

﻿﻿Jorge AA, Vanak AT, Thaker M, Begg CM, Slotow R. Costs and benefits of the presence of leopards to the sport-hunting industry and local communities in Niassa National Reserve, Mozambique. Conservation Biology. 2013;27: 832–843.

Lindsey P, Bento C. Illegal Hunting and the Bushmeat Trade in Central Mozambique: A Case-study From Coutada 9. Manica Province, Mozambique; 2012. <https://biblioteca.biofund.org.mz/wp-content/uploads/2019/01/1548835124-F0955.Illegal%20hunting%20and%20the%20bushmeat%20trade%20in%20Central%20Mozambique%20(PDF,%203%20MB).pdf>

﻿Lindsey PA, Balme G, Becker M, Begg C, Bento C, Bocchino C et al. The bushmeat trade in African savannas: Impacts, drivers, and possible solutions. Biological Conservation. 2013;160: 80–96.

Massé F, Lunstrum E. Accumulation by securitization: commercial poaching, neoliberal conservation, and the creation of new wildlife frontiers. Geoforum. 2016;69: 227–237.

Maugham R. Wild game in Zambezia. John Murray; 1914.

Nams VO, Parker DM, Weise FJ, Patterson BD, Buij R, Radloff FGT et al. Spatial patterns of large African cats: a large-scale study on density, home range size, and home range overlap of lions *Panthera leo* and leopards *Panthera pardus*. Mammal Review. 2023;53: 49–64.

Niassa Carnivore Programme. Annual report 2023. Niassa, Mozambique; 2023. <https://niassalion.org/wp-content/uploads/2024/07/2023-NCP-Annual-Report.pdf>

Norton P. How many leopards? A criticism of Martin and de Meulenaer's population estimates for Africa. South African Journal of Wildlife Research. 1990;86: 218–220.

﻿﻿Peace Parks Foundation. The story of a lion rescue in Zinave National Park; 2024. Available at: <https://www.peaceparks.org/africas-silent-killer-thwarted/>

Pringle RM. Upgrading protected areas to conserve wild biodiversity. Nature. 2017;546: 91–99.

Riggio J, Jacobson A, Dollar L, Bauer H, Becker MS, Dickman AJ et al. The size of savannah Africa: A lion’s (*Panthera leo*) view. Biodiversity and Conservation. 2012;22: 17–35.

Roque DV, Macandza VA, Zeller U, Starik N, Göttert T. Historical and current distribution and movement patterns of large herbivores in the Limpopo National Park, Mozambique. Frontiers in Ecology and Evolution. 2022;10.

Smithers RHN, Lobão Tello JLP. Checklist and atlas of the mammals of Mocambique. Museum Memoir No. 8, National Museums and Monuments of Rhodesia; 1976.

Smuts GL. Population characteristics and recent history of lions in two parts of the Kruger National Park. Koedoe. 1978;19: 153–164.

Stalmans ME, Massad TJ, Peel MJS, Tarnita CE, Pringle RM. War-induced collapse and asymmetric recovery of large-mammal populations in Gorongosa National Park, Mozambique. PLoS ONE. 2019;14: e0212864.
