# Supplementary material for: Unsustainable anthropogenic mortality threatens the long-term viability of lion populations in Mozambique
Source: PLoS One. 2025 Jun 10;20(6):e0325745. doi: 10.1371/journal.pone.0325745 (PMC12151417; doi:10.1371/journal.pone.0325745)
Supplement: Attachment — Submitted filename: Comments to the authors.docx [file pone.0325745.s002.docx]

**Comments to the authors on manuscript titled “Unsustainable anthropogenic mortality threatens the long-term viability of lion populations in Mozambique”**

I thoroughly enjoyed reading this manuscript. It is a compelling, well-written, and a highly relevant contribution to lion conservation. The authors present a clear and rigorous analysis of a pressing issue, and the integration of field data with modelling provides valuable insights for conservation policy and practice across Mozambique and beyond. The attention to regional variation and the nuanced discussion of anthropogenic pressures are particularly noteworthy. That said, the manuscript is very long and could benefit from being made more concise. Streamlining certain sections—particularly the Introduction and Discussion—would improve readability and sharpen the paper’s overall impact without compromising the quality of the work.

Apart from reducing the length I only have minor comments to address, mostly to improve grammar and flow.

Line 54 and 55: Remove the repeated use of “and” inserting commas instead, just keep ”and” for the end statement “and human-wildlife conflict”.

Line 65: Spell out IUCN on its first mention

Line 82: The word gazetted does not seem to fit well here, perhaps replace with a different word

Line 85: break this statement into two sentences as it is very long. I suggest putting a full stop after “resident communities”.

Lines 97-100: Consider adding a statement explaining why lions are being targeted for their body parts, for readers who may be unaware of why this trade exists.

Line 133: Change “gazetted” to Prioritized or something similar

Line 161: Just state that you “quantified the longitudinal impact.....” . There should not be aims mentioned in the methods, this kind of statement is suited for the introduction.

Lines 175-194. There are a lot of acronyms added in this section, and throughout the paper in general. While acronyms are useful for cutting down repeated words, in some cases it becomes confusing for the reader to keep track. I suggest cutting back on some of the acronyms, particularly in this section.

Line 196: Remove the word “the” after differences

Line 234-235: This statement is a little confusing, do you mean a daily average, an annual average or just the total average across the entire time frame?

Line 254: Change “towards” to “for”, it reads better.

Line 269: Also state what DCA is, again too many acronyms to keep track of.

Line 379: Can you provide any more information (perhaps this is better suited in the methods) as to how and when agencies could assign mortalities to targeted poaching for the IWT rather just opportunistic when lions were killed for other reasons? I realize how inherently difficult this might be, but is it just the case then when body parts are removed it is always assigned to poaching for IWT?

Line 386-389: This sentence is quite the mouthful and does not flow or read well, please revise.

Line 400: Insert “of” before “Limpopo

Lines 454-456: Do we know what is driving this increase in demand? Is the buyers market growing or are perhaps other previously sought after species for the IWT more difficult to obtain leading to a shift towards lions? Perhaps you add a statement and reference to other studies if anything is known about changes to this market

Line 467: I think you should add a statement to explain what this new legislation means for lion conservation in the country

Lines 474-478: This sentence is too long, break it up into two statements to improve flow.

Line 499: Inset “on” before ANAC

**Figures**

Figure 3: This figure is too chaotic and difficult for a reader to easily understand what is going on. I suggest this is changed to display the information in a more reader friendly format, even if this means breaking it up into a few smaller figures.

Figure 5 & 6: Are quite small and the legends seem a little bit blurry, please revise.
