# Supplementary material for: Unsustainable anthropogenic mortality threatens the long-term viability of lion populations in Mozambique
Source: PLoS One. 2025 Jun 10;20(6):e0325745. doi: 10.1371/journal.pone.0325745 (PMC12151417; doi:10.1371/journal.pone.0325745)
Supplement: Attachment — Submitted filename: Response to Reviewers.docx [file pone.0325745.s004.docx]

11 May 2025

Dear Dr Francesco Bisi and *PLoS ONE* Editorial board,

Thank you for considering this revised version of our manuscript entitled: “**Unsustainable anthropogenic mortality threatens the long-term viability of lion populations in Mozambique**” for publication in *PLoS ONE* as a Research Article. We are glad that it has been favourably reviewed and are happy to deal comprehensively with these major revisions as requested.

As ever, we appreciate peer-reviewed support for the conservation value and future application of our work. Overall, we have no major disagreements with the Editor or Reviewers, and as such, all suggested changes have been implemented, and clarification provided where requested or relevant given these corrections, as well as any explanation of statements and amendments requested. At the recommendation of Reviewers or to improve narrative flow, the following specific major changes have been made throughout the manuscript:

1) The Reviewers expressed concerns regarding overall length of the manuscript, particularly within the Introduction and the Discussion sections, and recommended that these parts be condensed. We fully acknowledge the importance of presenting scientific findings in a manner that is both comprehensive and succinct, to facilitate clarity and accessibility for readers. Given the interdisciplinary nature of our study, focusing on a nationally-scaled assessment of an endangered large carnivore, some degree of complexity and detail was necessary to adequately contextualize and support our findings. However, we understand that excessive length can obscure the overall trends and core message. In response, we have revised and streamlined the Introduction and Discussion to improve readability and enhance the overall flow of the manuscript.
2) The Editor and Reviewers also rightly raised the issue of consistent monitoring effort, referring both to population and mortality estimation, the effects of detectability bias and clarity regarding the classification of illegal wildlife trade based on the evidence presented. These are exceptionally valid considerations and present several limitations to analytical approaches and interpretation. We have addressed the concern regarding consistent monitoring effort and how this may have limited the early estimates of population size and mortality but highlight that this is less of a concern as robust monitoring and law enforcement efforts were rolled out for lion populations throughout the country. As suggested, these metrics are also highly susceptible to detectability biases, which we were able to retroactively correct for in the population estimates, but remain a limitation in quantifying mortality, though we justify our interpretation of overall trends by considering detected fluctuations in these data, which would be inconsistent with on ongoing detectability bias. We believe that accounting for these two aspects in this manner has allowed for a relatively consistent and robust interpretation of threat context to lion populations in Mozambique. We also offer improved clarity on our classification of illegal wildlife trade evidence throughout the revised manuscript, highlighting that ANY unpermitted killing of lions and subsequent possession or exchange of removed bodily parts constitutes illegal wildlife trade in Mozambique. We note however that the subtlety is in the targeted/syndicated versus opportunistic nature of such actions which is incredibly complex to analyse in a robust manner – while we have now indicated this as a consideration in the revised manuscript, it remains beyond the arguably overstretched but comprehensively valuable scope of the current manuscript.

We address the Editor’s comments regarding the journal requirements here. 1) We believe that our manuscript meets PLOS ONE's style requirements and are happy to make any additional changes if there are any inconsistencies. 2) The source data for this study are owned by each range state and can be accessed upon reasonable request via the IUCN/SSC Cat Specialist Group’s African Lion Database. 3) The ethics statement is included in the methods sections of the revised manuscript, along with an ethics statement, and 4) The globe presented in Figure 1 was obtained from Wikipedia under a Creative Commons license and manually edited, the country shapefiles were obtained from DivaGIS where it is freely available online, ArcGIS Pro and the remaining figures were created by the authors of this manuscript in Microsoft software.

This has been a highly informative and productive review for us as authors, thank you so much for the incredible support and efforts from the *PLoS ONE* team all around. We sincerely believe that the detailed comments and suggestions have substantially elevated the quality of this revised manuscript and are therefore not only grateful to the Reviewers for their time and consideration, but also to Dr Bisi for insights and kind support throughout this relatively rapid turn-around editing process.

Reviewer comment responses are included here (below), including point-by-point responses to the comments, and detailed rebuttals of any criticisms or requested revisions, on which we may have disagreed. We have incorporated these into the manuscript as best we can and have indicated where specific changes have been made (i.e., with highlighting and line references throughout – please note that all line references below refer to those in revised manuscript with track changes).

Thank you for your time and consideration, it is greatly appreciated,

Willem D. Briers-Louw

On behalf and with the consenting support of the author team.

PLOS ONE

Commentary and Revision

Decision on Manuscript ID: PONE-25-03127

## EDITORIAL COMMUNICATION:

Dear Dr. Briers-Louw,

Thank you for submitting your manuscript to PLOS ONE. After careful consideration, we feel that it has merit but does not fully meet PLOS ONE’s publication criteria as it currently stands. Therefore, we invite you to submit a revised version of the manuscript that addresses the points raised during the review process.

Please submit your revised manuscript by Jun 16 2025 11:59PM. If you will need more time than this to complete your revisions, please reply to this message or contact the journal office at [plosone@plos.org](mailto:plosone@plos.org). When you're ready to submit your revision, log on to <https://www.editorialmanager.com/pone/> and select the 'Submissions Needing Revision' folder to locate your manuscript file.

Please include the following items when submitting your revised manuscript:

- A rebuttal letter that responds to each point raised by the academic editor and reviewer(s). You should upload this letter as a separate file labelled 'Response to Reviewers'.
- A marked-up copy of your manuscript that highlights changes made to the original version. You should upload this as a separate file labelled 'Revised Manuscript with Track Changes'.
- An unmarked version of your revised paper without tracked changes. You should upload this as a separate file labelled 'Manuscript'.

If you would like to make changes to your financial disclosure, please include your updated statement in your cover letter. Guidelines for resubmitting your figure files are available below the reviewer comments at the end of this letter.

If applicable, we recommend that you deposit your laboratory protocols in [protocols.io](http://protocols.io) to enhance the reproducibility of your results. Protocols.io assigns your protocol its own identifier (DOI) so that it can be cited independently in the future. For instructions see: [https://journals.plos.org/plosone/s/submission-guidelines#loc-laboratory-protocols](https://track.editorialmanager.com/CL0/https:%2F%2Fjournals.plos.org%2Fplosone%2Fs%2Fsubmission-guidelines%23loc-laboratory-protocols/1/010f019691a8f06f-5598ef23-9c88-4479-8a1d-a1dcbbc00751-000000/UbAFXb0xZIy1Sxw7lh8v94Ysn3OEyVEq3SRsaSEhw-0=209). Additionally, PLOS ONE offers an option for publishing peer-reviewed Lab Protocol articles, which describe protocols hosted on [protocols.io](http://protocols.io). Read more information on sharing protocols at [https://plos.org/protocols?utm_medium=editorial-email&utm_source=authorletters&utm_campaign=protocols](https://track.editorialmanager.com/CL0/https:%2F%2Fplos.org%2Fprotocols%3Futm_medium=editorial-email%26utm_source=authorletters%26utm_campaign=protocols/1/010f019691a8f06f-5598ef23-9c88-4479-8a1d-a1dcbbc00751-000000/ooWE4_JnOGGyOsPayMea3EkV95Wi2sP251Lx2F7iYVA=209).

We look forward to receiving your revised manuscript.

Kind regards,

Francesco Bisi, Ph.D.
Academic Editor
PLOS ONE

------------------------------------------------------------------------------------------------------------------------------------

## Journal Requirements:

When submitting your revision, we need you to address these additional requirements.

1. Please ensure that your manuscript meets PLOS ONE's style requirements, including those for file naming. The PLOS ONE style templates can be found at

<https://journals.plos.org/plosone/s/file?id=wjVg/PLOSOne_formatting_sample_main_body.pdf> and

[https://journals.plos.org/plosone/s/file?id=ba62/PLOSOne_formatting_sample_title_authors_affiliations.pdf](https://track.editorialmanager.com/CL0/https:%2F%2Fjournals.plos.org%2Fplosone%2Fs%2Ffile%3Fid=ba62%2FPLOSOne_formatting_sample_title_authors_affiliations.pdf/1/010f019691a8f06f-5598ef23-9c88-4479-8a1d-a1dcbbc00751-000000/4vrsZwHjRaVn9w1RzSrRnJMZkZ4M74LZ2az4v7lqiR0=209)

2. We note that your Data Availability Statement is currently as follows: All relevant data are within the manuscript and in Supporting Information files.

Please confirm at this time whether or not your submission contains all raw data required to replicate the results of your study. Authors must share the “minimal data set” for their submission. PLOS defines the minimal data set to consist of the data required to replicate all study findings reported in the article, as well as related metadata and methods (<https://journals.plos.org/plosone/s/data-availability#loc-minimal-data-set-definition>).

For example, authors should submit the following data:

- The values behind the means, standard deviations and other measures reported;

- The values used to build graphs;

- The points extracted from images for analysis.

Authors do not need to submit their entire data set if only a portion of the data was used in the reported study.

If your submission does not contain these data, please either upload them as Supporting Information files or deposit them to a stable, public repository and provide us with the relevant URLs, DOIs, or accession numbers. For a list of recommended repositories, please see <https://journals.plos.org/plosone/s/recommended-repositories>.

If there are ethical or legal restrictions on sharing a de-identified data set, please explain them in detail (e.g., data contain potentially sensitive information, data are owned by a third-party organization, etc.) and who has imposed them (e.g., an ethics committee). Please also provide contact information for a data access committee, ethics committee, or other institutional body to which data requests may be sent. If data are owned by a third party, please indicate how others may request data access.

3. Your ethics statement should only appear in the Methods section of your manuscript. If your ethics statement is written in any section besides the Methods, please delete it from any other section.
4. We note that Figures 1-4 in your submission contain map/satellite images which may be copyrighted. All PLOS content is published under the Creative Commons Attribution License (CC BY 4.0), which means that the manuscript, images, and Supporting Information files will be freely available online, and any third party is permitted to access, download, copy, distribute, and use these materials in any way, even commercially, with proper attribution. For these reasons, we cannot publish previously copyrighted maps or satellite images created using proprietary data, such as Google software (Google Maps, Street View, and Earth). For more information, see our copyright guidelines: <http://journals.plos.org/plosone/s/licenses-and-copyright>.

We require you to either (1) present written permission from the copyright holder to publish these figures specifically under the CC BY 4.0 license, or (2) remove the figures from your submission:

1. You may seek permission from the original copyright holder of Figures 1-4 to publish the content specifically under the CC BY 4.0 license.

 We recommend that you contact the original copyright holder with the Content Permission Form (<http://journals.plos.org/plosone/s/file?id=7c09/content-permission-form.pdf>) and the following text:

“I request permission for the open-access journal PLOS ONE to publish XXX under the Creative Commons Attribution License (CCAL) CC BY 4.0 (<http://creativecommons.org/licenses/by/4.0/>). Please be aware that this license allows unrestricted use and distribution, even commercially, by third parties. Please reply and provide explicit written permission to publish XXX under a CC BY license and complete the attached form.”

Please upload the completed Content Permission Form or other proof of granted permissions as an "Other" file with your submission.

In the figure caption of the copyrighted figure, please include the following text: “Reprinted from [ref] under a CC BY license, with permission from [name of publisher], original copyright [original copyright year].”

2. If you are unable to obtain permission from the original copyright holder to publish these figures under the CC BY 4.0 license or if the copyright holder’s requirements are incompatible with the CC BY 4.0 license, please either i) remove the figure or ii) supply a replacement figure that complies with the CC BY 4.0 license. Please check copyright information on all replacement figures and update the figure caption with source information. If applicable, please specify in the figure caption text when a figure is similar but not identical to the original image and is therefore for illustrative purposes only.

The following resources for replacing copyrighted map figures may be helpful:

 USGS National Map Viewer (public domain): <http://viewer.nationalmap.gov/viewer/>

The Gateway to Astronaut Photography of Earth (public domain): <http://eol.jsc.nasa.gov/sseop/clickmap/>

Maps at the CIA (public domain): <https://www.cia.gov/library/publications/the-world-factbook/index.html> and <https://www.cia.gov/library/publications/cia-maps-publications/index.html>

NASA Earth Observatory (public domain): <http://earthobservatory.nasa.gov/>

Landsat: <http://landsat.visibleearth.nasa.gov/>

USGS EROS (Earth Resources Observatory and Science (EROS) Center) (public domain): [http://eros.usgs.gov/#](http://eros.usgs.gov/)

Natural Earth (public domain): <http://www.naturalearthdata.com/>

-------------------------------------------------------------------------------------------------------------------------

## EDITOR COMMENTS:

Dear Authors,

I had the pleasure to read you manuscript and I think it is very interesting, and data deserve to be shared with the public. I think it is important to have a better view of the conservation status of the lion in Africa and specifically in Mozambique and your manuscript could add an important contribution.

One of the weakest point is that you included so many information that sometimes it is not easy to understand them for a broader readership. I suggest shortening some parts as suggested by reviewer and try to be more focused on the hypotheses.

Please carefully follow reviewer’s suggestions.

We sincerely appreciate the Editor’s interest and support for this manuscript, highlighting its value both in the local context, but also for the conservation of the species more generally. We completely recognise the Editor’s concerns regarding the volume of information and the complexity it presents in a relatively brief contextual space. As such, we have attempted to shorten several sections and provided clarity throughout the revised manuscript that is now more focussed around the established hypotheses as suggested by the Editor and some of the Reviewers.

### 01 – E01|C01 (COMMENT):

To help a broader readership understand the problem, I think it would be helpful to clarify how you define distinct lion populations. Is the term population appropriate in all cases? For example, when there are only a few individuals, such as the two lions shown in Figure 1, can that be considered a population, or would pride be a more accurate term?

### 01 – E01|R01 (RESPONSE):

We appreciate this insightful and valuable comment from the editor. While fully support the notion of ensuring that scientific work is clear and can be understood by a broader readership, the notion of populations being ‘a demographically cohesive group of organisms of the same species occupying the same geographic area’ is generally open to several interpretations, depending on the context and scientific discipline. We also note that these populations, be they resident or transient, regardless of size (which is more likely an indication of limited monitoring or fragmented habitat which still supports transient corridors), are formally recognised by national and international policy. Even if these social groups were not defined as populations, their current use serves for the comparative purposes of the manuscript. Nevertheless, we are more than happy to provide a term to define distinct lion populations:

Line 180–184:	“In particular, we investigate comparatively large and well-monitored resident lion populations (i.e., a demographically cohesive group of lions occupying the same geographic area) occurring in Niassa SR, Gorongosa NP, Tchuma Tchato Community Programme, Zambezi Delta, Coutada 9 and 13 (hereafter, Coutadas 9/13), and Limpopo NP (Fig 1).”

### 02 – E01|C02 (COMMENT):

In Supplementary Material 1, you describe how the population in Coutadas 9/13 was extirpated, followed by reintroduction efforts, and that a hunting quota of two lions per year has been established. However, Figure 1 reports a population estimate of only two lions.

### 02 – E01|R02 (RESPONSE):

We thank the Editor for picking up on what, at face value, presents as an inconsistency in the logic around how populations and their quota offtake are presented. In reality, this is an honest indication of what we know about these Coutadas. Figures 1 and 2 show these adjacent Coutadas as having an estimated 44 lions in Coutada 9 and 2 lions in Coutada 13. While these populations are likely contiguous, their perceived threat, as well as resources and capacity to manage threats are reportedly different, which is an aspect which we wanted to bring to the fore. Importantly, it should also be noted that Coutada 13 is not currently under any form of formalised management and has not been for some time, which likely accounts for these low and likely under-represented population estimates – we have now provided this context in the revised Supplementary Materials as follows:

Line 056–084:	“The lion population in Coutadas 9 and 13 was severely depleted due to decades of armed conflict and persistent bushmeat poaching (Lindsey & Bento, 2012). While occasional sightings of transient individuals were reported, by the late 2000s, the population was considered functionally extirpated. In 2010, management of Coutada 9 initiated the reintroduction of ten lions with the aim of facilitating population recovery and generating additional revenue to sustain the WMAs. These lions, sourced from a private game reserve in South Africa, where they occurred in surplus, were considered ideal candidates for reintroduction. Following the reintroduction, the population displayed initial population growth, supported by abundant prey and a reduction in bushmeat poaching to more manageable levels (Lindsey & Bento, 2012). Regular monitoring of the population, including the use of satellite collars, and veterinary interventions were conducted in response to lions caught in steel gin (or ‘bear-claw’) traps, the main form of poaching in these WMAs (Lindsey et al., 2013), helped to enhance survival rates. Today, the population remains under pressure largely due to rising incidents of lion mortalities, primarily linked to bushmeat by-catch, with some evidence of targeted poaching for body parts. Coutada 9 is allocated a quota of two lions annually for trophy hunting, although actual offtake is occasionally lower than quota (e.g., in 2024 where only one lion was taken). This relatively small, regulated offtake helps to generate an essential proportion (~8%) of the total revenue for this WMA, which is critical for funding anti-poaching efforts and broader conservation efforts (Lindsey & Bento, 2012). In contrast, Coutada 13, adjacent to Coutada 9, has not been formally managed for several years due to complications around government-lease agreements, a situation mirrored by other vacant concessions across the country. Despite this lack of formal management, Coutada 13 remains an important area for lion conservation, both for its large size and for its role in supporting seasonal movements of lion and their prey from Coutada 9. Given the uncontrolled expansion of human settlements within Coutada 13, it is imperative that this WMA undergoes immediate and effective management to preserve this crucial habitat for lions and their prey. Without such intervention, the area risks becoming a sink for the lion population, undermining broader recovery efforts.”

While these two concessions are geographically adjacent, they differ substantially in context. Coutada 9 benefits from active management and ongoing protection efforts, while Coutada 13 has experienced periods of vacancy and poor management. It is also important to note that management in Coutada 9 reintroduced lions (i.e., 10 lions in 2010), not Coutada 13. In the Supplementary Material (Appendix S1), we state that “Coutada 9 is allocated a quota of two lions annually for trophy hunting”, which refers specifically to Coutada 9 that is actively managed and not Coutada 13. Currently, lions are not well monitored in Coutada 13, and we acknowledge that the estimate of two lions, which is rather typical of transient/vagrant-type populations, is not necessarily representative of the estimated lion population, but this is the best available information from this particular area. We acknowledge that it is difficult to know whether to split these two concessions or keep them as a single population. When considering Figure 2 in the manuscript, which addresses the perceived threat and management capacity indices, we thought it prudent to split Coutada 9 and 13 into two areas given the substantially higher threat indices reported for Coutada 13 compared to that of Coutada 9. When considering the size of these populations, we indicate in Figure 1, the number of lions in each of Coutada 9 and Coutada 13 to emphasize where the majority of the lions occur. However, given there are no fences and lions can move freely between these two areas, we considered these populations a single contiguous population for the forward simulation modelling to incorporate this. We also highlight that just because a quota is given for lion hunting, this full permit capacity is not always used and that we consider Coutada 13 a transient population (see Table 1 for further details).

### 03 – E01|C03 (COMMENT):

It would be important to also describe the structure of the survey used to generate the ATI and RCI indices, including at least the number of responses collected.

### 03 – E01|R03 (RESPONSE):

We thank the Editor for their suggestion to include detail on the survey structure used to generate the ATI and RCI indices – this is critical context that has now been included in the revised manuscript as suggested:

Lines 231–236:	“Surveys were completed at a subpopulation level, which we define as the non-transboundary lion area that is clearly separated by either political or formal protection boundaries [47]. One completed survey represented one lion subpopulation. We targeted managers of lion subpopulations within formal protected areas, recognised conservancies and game management areas, hunting concessions, and other wildlife areas. A total of 187 experts were invited to participate in the survey, of which 132 responded, yielding a response rate of 71%.”

### 04 – E01|C04 (COMMENT):

As you mention, data on lion mortalities are incomplete, since not all deaths are detectable. I'm curious whether the reporting effort remained constant throughout the study period, or if it might have varied due to awareness campaigns or other dedicated projects.

### 04 – E01|R04 (RESPONSE):

Thank you for highlighting a very important concern and certainly a contextual limitation to the work throughout. We fully agree that consistent detection of lion mortalities is challenging, and we acknowledge that it is almost impossible to fully control for, given the nature of these types of data. However, what we can confidently state is that both detectability and the quality of follow-up reporting have improved over time, particularly as a result of large-scale systematic law enforcement efforts as well as co-ordinated and systematic data capture the IUCN/SSC African Lion Database. While we cannot retroactively adjust for earlier under-detection, it is notable that mortality has continued to rise amid this known improvement in detection capacity. This suggests that a false-negative effect may have been more pronounced prior to 2013–2015, but since then, detection efforts, particularly those paired with follow-up investigations and reporting, have been relatively consistent. The ability to track fluctuations over time, even if imperfect, thus implies some degree of sampling saturation. If detectability had not plateaued, we would expect to see a continued upward trend in detection, not just these reported fluctuations. These challenges are inherent in this form of data, though we note that Mozambique maintains a relatively robust reporting system throughout the study period. That said, we fully recognize the Editor’s point and have incorporate this limitation more clearly in the revised manuscript:

Lines 544–556:	“Fourthly, efforts to monitor lion mortality and indicators of specific threats (e.g., lion conflict events, anti-poaching coverage, and snares removed) vary substantially across landscapes and are dependent on various factors such as protected area size and accessibility, as well as monitoring capacity and efficacy [13,62], resulting in variable detection rates and therefore confidence in projections [7,26,40,55]. Finally, while the number of reported lion mortalities has fluctuated over time, some of this variability may be attributed to seasonal accessibility or temporal variability in retaliatory killing, as well as demand for bushmeat or lion body parts for trade [13,62]. In addition, the detection of lion mortality may also be masked by cryptic poaching (i.e., poachers concealing their activities and avoiding detection) in response to increased antipoaching efforts [63]. In contrast, legal trophy hunting is comparatively well documented and regulated, thus these lion mortalities are likely near complete records [46]. Nevertheless, such regional population trend modelling accounts for such variability and provides important insights into the impact of this pervasive threat to lions across Mozambique.”

------------------------------------------------------------------------------------------------------------------------------------

## REVIEWER: 1

## General comments:

### 05 – R01|C01 (COMMENT):

The authors sought to measure the long-term effects of anthropogenic mortality on Mozambican lion populations in their work "Unsustainable anthropogenic mortality threatens the long-term viability of lion populations in Mozambique." The conclusions derived from this well-structured manuscript are consistent with the findings. I aim to offer some very helpful recommendations to raise the general level of clarity in your research and the calibre of your analysis. I believe you will be able to handle my recommendations, and I think they seem doable to you.

### 05 – R01|R01 (RESPONSE):

We sincerely thank the Reviewer for their thoughtful and generous comment. We also greatly appreciate their valuable recommendations to enhance the overall quality of the manuscript. In the responses below, we have endeavoured to address each of the Reviewer’s points as thoroughly and carefully as possible – thank you.

## Specific comments:

### 06 – R01|C02 (COMMENT):

Lines 62–119: Please reduce this part of the manuscript.

### 06 – R01|R02 (RESPONSE):

We appreciate the Reviewer’s regarding the length of this particular section of the Introduction. We do feel that there are fine margins between keeping the Introduction brief and potentially not introducing important aspects for context to the manuscript as a whole and including all information for the readers to fully understand, but then potentially missing the main focus of the paper. Nevertheless, we had followed the Reviewers suggestion and trimmed parts of the Introduction (Lines 70–75) to ensure the focus of the manuscript is clearly visible. Below are a few examples of where we have reduced the text, as suggested:

Lines 70–75:	“These range states are often unable to keep pace with mounting anthropogenic threat levels, experiencing a ~60% decline over the same period, with a notable increase in anthropogenic mortalities [18]. ~~For instance, rampant bushmeat snaring likely led to the extirpation of lions in the Nsumbu National Park (NP) of Zambia (Dröge pers comm., 2022), while the population in Limpopo NP of Mozambique has shown severe risk of collapse due to HWC and increased targeted poaching pressure for IWT [23,36].~~ Unfortunately, similar declines are predicted for many lion populations across their African range, especially in the most ecologically and socio-politically fragile countries [24,25], which may be without adequate resources and management capacity to ameliorate these threats [18,26].“

Lines 92–94:	“However, in the shadow of historical armed conflict and political instability ~~(i.e., 1964–1975, 1977–1992)~~, Mozambican conservation continues to grapple with ongoing localized conflict, human settlement within or around PAs and unprecedented growth of resident communities.”

Lions 97–98:	“Lions currently occur in six resident populations across Mozambique ~~(i.e., Niassa Special Reserve [SR], Gorongosa NP, Tchuma Tchato Community Programme (TTCP), Zambezi Delta, Coutada 9 and 13 (hereafter Coutadas 9/13) and Limpopo NP)~~.”

### 07 – R01|C03 (COMMENT):

Lines 120–129: Please explain in detail your hypothesis and predictions. You need to expand this section if you would want to express exactly what you want to do.

### 07 – R01|R03 (RESPONSE):

We sincerely thank the Reviewer for their suggestion of explaining our hypotheses and predictions in the aims section of the Introduction. We agree this would be a valuable addition to improve the quality and overarching direction of the revised manuscript:

Lines 153–157:	“We hypothesise that the proportion of anthropogenically linked lion mortality in Mozambique directly threatens the long-term viability of resident populations. We also predict that relative protected area size and perceived threat, as well as management resources and capacity contribute to the resilience of lion these populations. While the targeted poaching of lions for the illegal wildlife trade significantly contributes to resident lion extirpation risk.”

### 08 – R01|C04 (COMMENT):

Lines 196–227: The authors must add all the R codes used in this study in the supplementary materials. Please add R codes well described and understandable.

### 08 – R01|R04 (RESPONSE):

We acknowledge the Reviewer’s recommendation. The R source code is now available in a FigShare repository (<https://doi.org/10.6084/m9.figshare.29040002>) – thank you.

### 09 – R01|C05 (COMMENT):

Line 343: Please DELETE all the references from the results section.

### 09 – R01|R05 (RESPONSE):

We appreciate the Reviewer’s comment. In the three instances references were included in the Results section of the manuscript, they were merely intended to cite the methodological components underpinning the respective findings. However, as these methodological details are already thoroughly addressed in the Methods section and figure captions, we have followed the Reviewer’s recommendation and removed these citations from the Results section in the revised manuscript.

### 10 – R01|C06 (COMMENT):

Lines 361-362: I think that you should add these two important references as examples to support your sentence: “Anthropogenic mortality has driven large carnivore population declines and extirpations across their ranges.”.

I would like to suggest:

Ahmad, F., et al., (2025). Asiatic black bear in Pakistan: a comprehensive review and conservation indications. Mammalian Biology. <https://doi.org/10.1007/s42991-025-00479-x>

Lohr, M. T., et al., (2025). Widespread detection of second generation anticoagulant rodenticides in Australian native marsupial carnivores. Science of the Total Environment, 967(17883), 178832.

### 10 – R01|R06 (RESPONSE):

We fully agree with the Reviewer that this particular sentence requires a reference for support. We also sincerely appreciate the suggested two references kindly provided by the Reviewer. Given that we have a number of references already cited in the manuscript, especially in the Introduction, which support this statement sufficiently, we have decided to use from these existing references to support the statement.

Lines 461–462:	“Anthropogenic mortality has driven large carnivore population declines and extirpations across their range [12].”

### 11 – R01|C07 (COMMENT):

Lines 365–508: This part of the manuscript should be reduced.

### 11 – R01|R07 (RESPONSE):

Given the interdisciplinary scope of our study, which entails a nation-wide assessment of lions, some degree of complexity and detail is essential to appropriately contextualize the research and substantiate the findings. We do however acknowledge that the Discussion section of this manuscript is rather long in certain parts. As a result, we take the Reviewer’s suggestion under consideration in our response, by clearly demonstrating sections that have been shortened in the text in the revised manuscript and elsewhere throughout. Please see examples below of where such reductions have been made:

Line 465–467:	“Quantification of anthropogenic mortality throughout post-war Mozambique revealed that bushmeat poaching, among other threats, is a significant suppressor of regional lion population recovery. ~~Furthermore, the predominant factors undermining system-wide resilience are relatively small lion populations and limited management capacity.~~”

Line 493–495:	“In Niassa SR, ~~the largest population (i.e., 4x larger than Limpopo NP) and lion stronghold (i.e., constituting~~which supports 62–77% of the national lion population~~) in Mozambique~~, ~~the~~ anthropogenic mortality rates range from ~~is~~ low to high (3.2–16%), with the precision of these estimates constrained by ~~and protracted, where~~ low detection rates (~20%) ~~limit the precision of these projections~~.”

Line 543–547:	“Fourthly, efforts to monitor lion mortality and indicators of specific threats (e.g., lion conflict events, anti-poaching coverage, and snares removed) vary substantially across landscapes and are dependent on various factors such as PA size and accessibility, as well as monitoring capacity and efficacy [13,62], resulting in variable detection rates and therefore confidence in projections [7,26,40,55]. ~~For instance, limited lion monitoring in certain areas of the country (e.g., the TTCP which receives the second largest annual hunting quota; CITES Trade Database: https://trade.cites.org/) resulted in data deficiencies which could not be used to model population size and anthropogenic mortality risk.~~”

Line 631–634:	“Similar ~~increases in lion poaching for body parts~~ trends have been recorded for Niassa SR since 2015 [39]. ~~Predicted population trends, given detectable anthropogenic mortality, in these two stronghold populations is of regional policy and management concern.~~ ~~Where Niassa SR,~~ This largest population in the country, remains under-resourced resulting in a poor detection of anthropogenic mortalities and is already at the projected threshold of unsustainable offtake.”

### 12 – R01|C08 (COMMENT):

Figures 1-4: Please add the north symbol and the scale in the map.

### 12 – R01|R08 (RESPONSE):

We appreciate the Reviewer’s attention to detail with regard to the lack of north arrows in each of the Figures 1–4. We have subsequently revised these Figures and included a north arrow in each as recommended by the Reviewer – thank you.

------------------------------------------------------------------------------------------------------------------------------------

## REVIEWER: 2

## General comments:

### 13 – R02|C01 (COMMENT):

I thoroughly enjoyed reading this manuscript. It is a compelling, well-written, and a highly relevant contribution to lion conservation. The authors present a clear and rigorous analysis of a pressing issue, and the integration of field data with modelling provides valuable insights for conservation policy and practice across Mozambique and beyond. The attention to regional variation and the nuanced discussion of anthropogenic pressures are particularly noteworthy. That said, the manuscript is very long and could benefit from being made more concise. Streamlining certain sections—particularly the Introduction and Discussion—would improve readability and sharpen the paper’s overall impact without compromising the quality of the work. Apart from reducing the length I only have minor comments to address, mostly to improve grammar and flow.

### 13 – R02|R01 (RESPONSE):

We thank the Reviewer for their generous and comprehensive feedback. We are grateful for their recognition of the considerable effort invested in this manuscript, and we sincerely hope that our work can contribute meaningfully to lion conservation in Mozambique and across Africa. We acknowledge the Reviewer’s concern regarding the length of the Introduction and Discussion – an issue that was also highlighted by another Reviewer – which underscores the need for refinement. In response, we have made a concerted effort to shorten these sections. Below, we address each Reviewer’s specific comments as thoroughly and thoughtfully as possible, in the hope that their concerns are sufficiently assuaged.

## Specific comments:

### 14 – R02|C02 (COMMENT):

Line 54 and 55: Remove the repeated use of “and” inserting commas instead, just keep ”and” for the end statement “and human-wildlife conflict”.

### 14 – R02|R02 (RESPONSE):

We thank the Reviewer for their suggestion to improve the readability and flow of this particular sentence. We have changed the sentence accordingly in the revised manuscript:

Lines 52–55:	“In Africa, anthropogenic threats to wildlife are severe and pervasive at the continental (e.g., land/sea use change, natural resource extraction ~~and~~, pollution, climate change [4,5]), regional (e.g., illegal wildlife trade [IWT] ~~and~~, poorly regulated legal harvest [6]), and local (e.g., poaching [7] and human-wildlife conflict [HWC; 8]) scales.”

### 15 – R02|C03 (COMMENT):

Line 65: Spell out IUCN on its first mention

### 15 – R02|R03 (RESPONSE):

We fully agree with the Reviewer that IUCN needs to be spelled out fully at first mention. We have made the necessary change in the revised manuscript as suggested:

Lines 64–68:	“Despite being listed as ‘Vulnerable’ globally (International Union for Conservation of Nature Red List of Threatened Species [17]), successful lion conservation resulted in a ~12% increase between 1993 and 2014 across Botswana, Namibia, South Africa and Zimbabwe, largely due to the proliferation of reintroductions into relatively small (i.e., <1,000 km^2^), fenced, and well-funded metapopulation reserves [18,22].”

### 16 – R02|C04 (COMMENT):

Line 82: The word gazetted does not seem to fit well here, perhaps replace with a different word

### 16 – R02|R04 (RESPONSE):

The term ‘gazetted’, in our opinion, is frequently used in the context of government formally setting aside land for conservation, such as national parks and national reserves. However, we are happy to change the term if the Reviewer does not feel it is an apt term in this context. We have decided on the term ‘designated’ – we hope this is applied satisfactorily:

Lines 90–91:	“With 29% of the country formally ~~gazetted~~designated for conservation [27], there is an opportunity for population recovery and range restoration.”

### 17 – R02|C05 (COMMENT):

Line 85: break this statement into two sentences as it is very long. I suggest putting a full stop after “resident communities”.

### 17 – R02|R05 (RESPONSE):

We fully agree with the Reviewer that this sentence is too long. We have followed the Reviewer’s suggestion and split this sentence into two in the revised manuscript:

Lines 92–96:	“However, in the shadow of historical armed conflict and political instability (i.e., 1964–1975, 1977–1992), Mozambican conservation continues to grapple with ongoing localized conflict, human settlement within or around PAs and unprecedented growth of resident communities~~,w~~. When coupled with severe and ongoing shortages in funding for PA management, these challenges have driven unsustainable levels of resource extraction [28,29].”

### 18 – R02|C06 (COMMENT):

Lines 97-100: Consider adding a statement explaining why lions are being targeted for their body parts, for readers who may be unaware of why this trade exists.

### 18 – R02|R06 (RESPONSE):

We thank the Reviewer for their valuable suggestion. We fully agree that including an explanation around why the trade in lion body parts exists is essential context for readers, who may be unaware of why this trade exists. Following the Reviewer’s suggestion, we have offered a sentence in the revised manuscript to briefly explain this particular issue. While we could easily expand on this particular sentence, we also consider the suggestions from another Reviewer to reduce the length of the Introduction. We hope this is satisfactory as presented below:

Lines 104–110:	“Mozambique also appears to have become a regional hotspot for IWT in lion derivatives, both as a source and transit state [35,36], that is particularly vulnerable to corruption and poor enforcement [14,37]. While the precise drivers of demand remain speculative, studies suggest that lion body parts are typically sought for use in zootherapeutic traditional medicine (i.e., zootherapy), and social or cultural purposes (e.g., clothing, curios, ornaments, trinkets, and other regalia) in both Asian and African markets [35,36,38]. Poaching of lions for body parts has thus raised significant concerns for lion conservation throughout the region [38,39].”

### 19 – R02|C07 (COMMENT):

Line 133: Change “gazetted” to prioritized or something similar.

### 19 – R02|R07 (RESPONSE):

We thank the Reviewer for the suggestion. We agree that ‘prioritized’ fits better in this particular context and have replaced these terms in the revised manuscript.

Lines 164–165:	“This study reports on longitudinal lion population and mortality data collected from various areas ~~gazetted~~prioritized for conservation in Mozambique (Table 1).”

### 20 – R02|C08 (COMMENT):

Line 161: Just state that you “quantified the longitudinal impact.....”. There should not be aims mentioned in the methods, this kind of statement is suited for the introduction.

### 20 – R02|R08 (RESPONSE):

We appreciate this suggestion by the Reviewer. We fully agreed that the term “aim” should be removed in this section. We have made the necessary change in the revised the manuscript:

Line 206–207:	“We ~~The study aimed to~~ quantif~~y~~ied the longitudinal impact of anthropogenic mortality on lion populations in Mozambique using national population estimates and monitoring records between 2010 and 2023.”

### 21 – R02|C09 (COMMENT):

Lines 175-194. There are a lot of acronyms added in this section, and throughout the paper in general. While acronyms are useful for cutting down repeated words, in some cases it becomes confusing for the reader to keep track. I suggest cutting back on some of the acronyms, particularly in this section.

### 21 – R02|R09 (RESPONSE):

We thank the Reviewer for their suggestion regarding the use of acronyms. We fully understand that the excessive use of acronyms may be a burden to readers, especially if missed early on in the manuscript. We have followed the Reviewer’s suggestion by removing several instances of acronyms, from this and other section of the manuscript. For example, we removed the following:

‘DCA’ – replaced with damage-causing animal

‘GLC’ – replaced with Greater Lebombo Conservancy

‘MUL’ – replaced with mixed-use landscape

‘TTCP’ – replaced with Tchuma Tchato Community Programme

‘WMA’ – replaced with wildlife management area

### 22 – R02|C10 (COMMENT):

Line 196: Remove the word “the” after differences.

### 22 – R02|R10 (RESPONSE):

Thank you for noticing this typographical error. We have removed this word in revised manuscript.

### 23 – R02|C11 (COMMENT):

Line 234-235: This statement is a little confusing, do you mean a daily average, an annual average or just the total average across the entire time frame?

### 23 – R02|R11 (RESPONSE):

We appreciate the Reviewer’s comment regarding the ambiguity of this particular statement. We fully agree and have revised this in the manuscript to make it clearer.

Lines 337–339:	“This constitutes an average of approximately 30 mortalities (i.e., ~2.3% of the total population) and 1.31 interventions (i.e., ~0.1% of the total population) per year (at a mean of 5.77 ± 2.67 [SE] lions per population).”

### 24 – R02|C12 (COMMENT):

Line 254: Change “towards” to “for”, it reads better.

### 24 – R02|R12 (RESPONSE):

We thank the Reviewer for their comment. We fully agree with this recommendation and have made the necessary change in the revised manuscript.

25 **– R02|C13 (COMMENT):**

Line 269: Also state what DCA is, again too many acronyms to keep track of.

### 25 – R02|R13 (RESPONSE):

We acknowledge this suggestion by the Reviewer, in addition to a previous comment (38 – R02|C09 (COMMENT) regarding the reduction of acronyms in the manuscript. We have changed “DCA” to “damage-causing animal” throughout the revised manuscript. Thank you for the suggestion.

### 26 – R02|C14 (COMMENT):

Line 379: Can you provide any more information (perhaps this is better suited in the methods) as to how and when agencies could assign mortalities to targeted poaching for the IWT rather just opportunistic when lions were killed for other reasons? I realize how inherently difficult this might be, but is it just the case then when body parts are removed it is always assigned to poaching for IWT?

### 26 – R02|R14 (RESPONSE):

We thank the Reviewer for this exceptionally valid point and completely agree with the underlying complexity in making this inference (please see major response 2 in the Cover Letter for further details). We have specifically offered improved clarity on our classification of illegal wildlife trade evidence throughout the revised manuscript, highlighting that ANY unpermitted killing of lions and subsequent possession or exchange of removed bodily parts constitutes illegal wildlife trade in Mozambique – while we have now indicated this as a consideration in the revised manuscript, it remains beyond the arguably overstretched but comprehensively valuable scope of the current manuscript:

Lines 219–222:	“Targeted poaching of lions was defined as an anthropogenic mortality with i) no evidence of HWC, ii) evidence of deliberate attempts to kill lions, and iii) body parts removed [38]. Due to the complex nexus of suspected motives, we could not reliably confirm this for all incidents of anthropogenic mortality from the information provided.”

We concede however that the subtlety is in the targeted/syndicated versus opportunistic nature of such actions which is incredibly complex to analyse in a robust manner. Where such analysis has been attempted before, conviction statements have been used to corroborate motives (also carrying inherent reporting biases) and/or multi-point behavioural pattern analyses have been used to link seemingly random (and therefore ‘opportunistic’) behaviour to poaching activities, versus co-ordinated actions (and therefore ‘targeted’ or intentional) actions with poaching events. These analyses have been successfully applied to complex scenarios such as rhino poaching in southern Africa, however, this requires extensive monitoring resources. Moreover, potential lion poachers in these Mozambican landscapes are not monitored, criminals are rarely convicted successfully and there is limited follow-up information regarding motive collected outside of the conviction itself.

### 27 – R02|C15 (COMMENT):

Line 386-389: This sentence is quite the mouthful and does not flow or read well, please revise.

### 27 – R02|R15 (RESPONSE):

We agree with Reviewer that this sentence contains quite a lot of information and may be difficult to process. In response, we have both restructured the sentence and removed portions of information (e.g. relative size of Niassa SR compared to other PAs in Mozambique) that can be obtained from the figures with the manuscript.

Line 493–495:	“In Niassa SR, ~~the largest population (i.e., 4x larger than Limpopo NP) and lion stronghold (i.e., constituting~~which supports 62–77% of the national lion population~~) in Mozambique~~, ~~the~~ anthropogenic mortality rates range from ~~is~~ low to high (3.2–16%), with the precision of these estimates constrained by ~~and protracted, where~~ low detection rates (~20%) ~~limit the precision of these projections~~.”

### 28 – R02|C16 (COMMENT):

Line 400: Insert “of” before “Limpopo

### 28 – R02|R16 (RESPONSE):

Thank you for picking up on the typographical error. We have added the word “of” before Limpopo in the revised manuscript as suggested by the Reviewer.

### 29 – R02|C17 (COMMENT):

Lines 454-456: Do we know what is driving this increase in demand? Is the buyer’s market growing or are perhaps other previously sought after species for the IWT more difficult to obtain leading to a shift towards lions? Perhaps you add a statement and reference to other studies if anything is known about changes to this market.

### 29 – R02|R17 (RESPONSE):

We thank the Reviewer for their insightful question regarding the increase in demand for lion bones and derivatives within the illegal wildlife trade (IWT). We found this point particularly valuable in improving the quality of the manuscript. It is indeed plausible that the observed change in demand reflects the emergence of a growing buyer and consumer market. However, disentangling whether this trend represents a direct increase in demand specifically for lions, or whether lions are increasingly being used as substitutes for more traditionally sought-after species, namely tigers, in the Asian markets remains complex. Tigers have become increasingly scarce in illegal markets due to their reduced global population (estimated at ~3,140 individuals; Goodrich et al., 2022) and increased protection efforts of the species across their range. This relative scarcity appears to have redirected demand toward African lions to fill that gap (Williams, 2015; Williams et al., 2017).

Studies report that lion imagery first began appearing on labels of Chinese medicines in 2005, with the first official CITES export permit for lion ‘skulls, skins and bones’ issued in 2008 (Williams et al., 2017). This marked the beginning of a sharp rise in the volume of lion body parts exported to Asia, as detailed in Lines 623-631 of the manuscript. Furthermore, the widespread use of Internet use, particularly through social media platforms, has also been reported to further enable IWT (including for lions), as it allows demand for specific species or body parts to be rapidly broadcast to wide audiences, potentially triggering sudden increases in consumer interest (Lavorgna, 2014).

Following the Reviewer’s suggestion, we have provided an explanation for this increase in trade of lion body parts, with associated references in the revised manuscript. We note that due to word count and suggested reductions by another reviewer, we purposefully keep this particular explanation relatively brief. However, we hope this addition suffices:

Lines 623-631:	“This declining population trend was matched by an increasing demand for lion body parts in the IWT, particularly from 2014 [38], alongside a near five-fold parallel increase in legal exports of lion skeletons from southern Africa to south-east Asia [36,61]. This is likely evidence of an expanding trader and consumer market, with lions increasingly used to supplement previously targeted species, especially tigers (*Panthera tigris*), which have become less accessible due to population declines and improved protection efforts in Asia [35]. Though such informed speculation should not be conflated with interpretation [56]. Such Asian influence in regional lion body part trade is not limited to end-user demand, rather, there is a chain of local exploitation and influence which feeds into syndicated international IWT, that is particularly pronounced in Mozambique [35,38].”

**References**

Arias M, Coals P, Ardiantiono, Elves-Powell J, Rizzolo JB, Ghoddousi A, et al. (2024) Reflecting on the role of human-felid conflict and local use in big cat trade. Conservation Science and Practice 6: e13030. https://doi.org/10.1111/csp2.13030

Goodrich J, Wibisono H, Miquelle D, Lynam AJ, Sanderson E, Chapman S, Gray TNE, Chanchani P, Harihar A (2022) Panthera tigris. The IUCN Red List of Threatened Species 2022: e.T15955A214862019. https://dx.doi.org/10.2305/IUCN.UK.2022- 1.RLTS.T15955A214862019.en

Lavorgna A (2014) Wildlife tracking in the Internet age. Crime Scene 3: 5.

Williams VL, Loveridge AJ, Newton DJ, Macdonald DW (2017) A roaring trade? The legal trade in *Panthera leo* bones from Africa to East-Southeast Asia. PLoS ONE 12(10): e0185996. <https://doi.org/10.1371/journal.pone.0185996>

Williams V (2015) Tiger-bone trade could threaten lions. Nature 523(7560): 290. <https://doi.org/10.1038/523290a>

### 30 – R02|C18 (COMMENT):

Line 467: I think you should add a statement to explain what this new legislation means for lion conservation in the country.

### 30 – R02|R18 (RESPONSE):

We appreciate the Reviewer’s suggestion to include a statement clarifying the implications of the new legislation in Mozambique for lion conservation. Broadly, and particularly in light of the elevated levels of lion poaching in the country, the Conservation Areas Law (Act No. 7 of 2017) introduces more stringent penalties for wildlife crimes (including poaching). In the case of lions – listed under Appendix II of CITES – the law imposes substantial fines of up to 1,000 times the national minimum wage and prison sentences of up to 12 years. While sentencing remains case-specific, it is hoped that the introduction of these harsher penalties will serve as a deterrent to potential offenders and contribute meaningfully to lion conservation efforts:

Lines 636–641:	“In an ~~attempt~~effort to combat wildlife-related crime ~~in~~, Mozambique~~,~~ ~~new conservation legislation has been passed~~enacted the ~~(i.e.,~~ Conservation Areas Law (Act Nº7 of 2017), which amends and strengthens provisions from ~~that introduce changes on the~~ Act 16 of 2014). This new legislation aligns with international conservation standards and imposes more stringent penalties for wildlife crimes, particularly those involving species, like lions, that are listed under the Convention on International Trade in Endangered Species of Wild Fauna and Flora, with prison sentences ranging up to 12 years.”

### 31 – R02|C19 (COMMENT):

Lines 474-478: This sentence is too long, break it up into two statements to improve flow.

### 31 – R02|R19 (RESPONSE):

In the lines 474–478, we attempted explain the importance of monitoring of lion populations and their threats collectively. However, we fully agree with the Reviewer that this particular sentence is long and, as a result, does not flow well. We have split this text into two sentences in the revised manuscript as recommended by the Reviewer – thank you:

Lines 647–652:	“To address these threats, we encourage improved monitoring of lion populations, allowing for greater responsiveness when problems arise~~,~~ and providing a stronger basis for ~~more~~evidence-based quota setting~~, while~~. Additionally, improved detection of anthropogenic mortality through a standardized monitoring system would help to generate more accurate data with which to assess ~~the~~impacts on lion population trends~~, as well as~~and evaluate the efficacy of current conservation interventions [40,70].”

### 32 – R02|C20 (COMMENT):

Line 499: Inset “on” before ANAC.

### 32 – R02|R20 (RESPONSE):

Again, thank you for noticing the typographical error. We really appreciate the attention to detail and are more than happy to make this change – it is now correctly reflected in the revised manuscript.

### 33 – R02|C21 (COMMENT):

Figure 3: This figure is too chaotic and difficult for a reader to easily understand what is going on. I suggest this is changed to display the information in a more reader friendly format, even if this means breaking it up into a few smaller figures.

### 33 – R02|R21 (RESPONSE):

We appreciate the Reviewer’s comment and suggestion to make the figure in question more reader-friendly. Our aim with Figure 3 was to visually depict the typology of anthropogenic lion mortality. However, we understand the concern raised by the Reviewer, and agree that splitting Figure 3 into smaller units may be easier from a readability perspective. We have followed the Reviewer’s suggestion by dividing Figure 3 into two figures – Figure 3 (a & b) have become Figure 3 (a & b), while Figure 3 (c & d) have become Figure 4 (a & b). We hope that these changes assuage the concerns presented by the Reviewer.

### 34 – R02|C22 (COMMENT):

Figure 5 & 6: Are quite small and the legends seem a little bit blurry, please revise.

### 34 – R02|R22 (RESPONSE):

We thank the Reviewer for noticing the low quality of Figures 5 and 6. We have improved the resolution of both figure in the revised manuscript. Please not that these figures are now Figures 6 and 7, due to the splitting of Figure 3 into Figures 3 and 4.

----------------------------------------------------------- END -----------------------------------------------------------
